# Deciphering the response of *Mycobacterium smegmatis* to nitrogen stress using bipartite active modules

**DOI:** 10.1186/1471-2164-14-436

**Published:** 2013-07-02

**Authors:** Kerstin J Williams, William A Bryant, Victoria A Jenkins, Geraint R Barton, Adam A Witney, John W Pinney, Brian D Robertson

**Affiliations:** 1 Department of Medicine, MRC Centre for Molecular Bacteriology and Infection, South Kensington, London SW7 2AZ, UK; 2 Centre for Integrative Systems Biology and Bioinformatics, Imperial College London, South Kensington, London SW7 2AZ, UK; 3 Centre of Infection and Immunity, St George's, University of London, SW17 0RE, London, UK

**Keywords:** Nitrogen Stress, Mycobacteria, Gene Expression, Metabolic Network

## Abstract

**Background:**

The ability to adapt to environments with fluctuating nutrient availability is vital for bacterial survival. Although essential for growth, few nitrogen metabolism genes have been identified or fully characterised in mycobacteria and nitrogen stress survival mechanisms are unknown.

**Results:**

A global transcriptional analysis of the mycobacterial response to nitrogen stress, showed a significant change in the differential expression of 16% of the *Mycobacterium smegmatis* genome. Gene expression changes were mapped onto the metabolic network using Active Modules for Bipartite Networks (AMBIENT) to identify metabolic pathways showing coordinated transcriptional responses to the stress. AMBIENT revealed several key features of the metabolic response not identified by KEGG enrichment alone. Down regulated reactions were associated with the general reduction in cellular metabolism as a consequence of reduced growth rate. Up-regulated modules highlighted metabolic changes in nitrogen assimilation and scavenging, as well as reactions involved in hydrogen peroxide metabolism, carbon scavenging and energy generation.

**Conclusions:**

Application of an Active Modules algorithm to transcriptomic data identified key metabolic reactions and pathways altered in response to nitrogen stress, which are central to survival under nitrogen limiting environments.

## Background

Nitrogen is a fundamental constituent of the bacterial cell, found in DNA, RNA, proteins and cell wall components, and its assimilation is consequently essential for bacterial growth. In order to cope with varying availability of nitrogen in the environment, bacteria have evolved a variety of mechanisms for the uptake of nitrogen from the environment, and its subsequent incorporation into biomass, all of which are tightly regulated [1,2]. Historically, mechanisms described in *Escherichia coli* have served as the model for nitrogen limitation [3]. However, more recent studies of two Actinomycetes (*Corynebacterium glutamicum* and *Streptomyces coelicolor*) have shown that the nitrogen control mechanisms in these bacteria not only differ from those in *E. coli,* but are distinct from each other [4-9]. It is clear that diverse strategies to respond to changes in nitrogen availability have evolved in the prokaryote kingdom.

*Mycobacterium smegmatis* contains the highest number of predicted nitrogen metabolism genes of any sequenced mycobacterial genome, many of which originate in other bacteria and have been acquired by horizontal transfer [10]. This fits with its saprophytic lifestyle, and the slow growing, pathogenic mycobacteria have all lost a proportion of their genes for nitrogen uptake and assimilation [10]. However the functions of most of these genes have not been verified experimentally and their roles in nitrogen assimilation and metabolism are currently unknown. Ammonium is the preferred nitrogen source for *M. smegmatis,* which contains 3 transporters (AmtA, AmtB and Amt1), but the genome also contains all the components necessary to obtain ammonium from other nitrogenous sources such as urea and nitrite. Once inside the cell ammonium is converted into glutamate and glutamine, the building blocks of most nitrogen containing compounds.

Modulation of gene expression plays a central role in cellular adaptation to environmental changes. Gene expression profiles have been obtained for mycobacteria exposed to numerous different stress conditions, including total nutrient starvation [11-16], but there is no reported global transcriptional analysis of the nitrogen stress response. In *E. coli* the nitrogen stress response consists of approximately 100 genes, mediated by the two-component signal transduction system NtrB/NtrC and regulatory protein Nac [3,17], but this system is not present in the Actinomycetes. In *S. coelicolor* the nitrogen response regulator is the transcriptional activator GlnR, which controls at least 50 genes [18-20]. *M. smegmatis* possesses a GlnR homologue (MSMEG5784), with 55% amino acid identity to the *S. coelicolor* GlnR, with 52 GlnR binding sites during nitrogen limitation controlling the expression of over 100 genes [21-23]. Therefore, in order to expand our knowledge of the genes involved in nitrogen metabolism and stress in mycobacteria, a global transcriptional profile of the nitrogen stress response is required.

Insights into the metabolic response of interest beyond those available through analysis of individual genes can be extracted from transcriptomic analyses through system level approaches. Enrichment analyses based on Fisher’s exact test [24] or Gene Set Enrichment Analysis [25] are often used to provide such system level insights, but are limited to predefined gene sets and pathways. More sophisticated methods for the analysis of metabolic changes, such as Differential Producibility Analysis (DPA) [26] and E-flux [27] are based on Flux Balance Analysis [28] and hence depend on a high quality, curated genome scale metabolic network, which is not currently available for *M. smegmatis*. However there is a freely usable online pipeline (Model SEED [29]) for the automatic production of draft quality metabolic models for any prokaryote with a complete genome sequence. We therefore used AMBIENT [30], which takes advantage of the network structure of metabolism to find non-predefined metabolic pathways affected by nitrogen limitation. Since AMBIENT does not use FBA, its results are not strongly dependent on the quality of the metabolic network, and the metabolic model produced by Model SEED for *M. smegmatis* can be used. AMBIENT was applied in this study to explore the system level adaptation of *M. smegmatis* to nitrogen starvation. This analysis revealed several key features of the metabolic response of *M. smegmatis* to nitrogen stress, including areas of metabolism not identified by KEGG enrichment analysis.

## Results and discussion

### Defining nitrogen stress conditions

**Figure 1:**
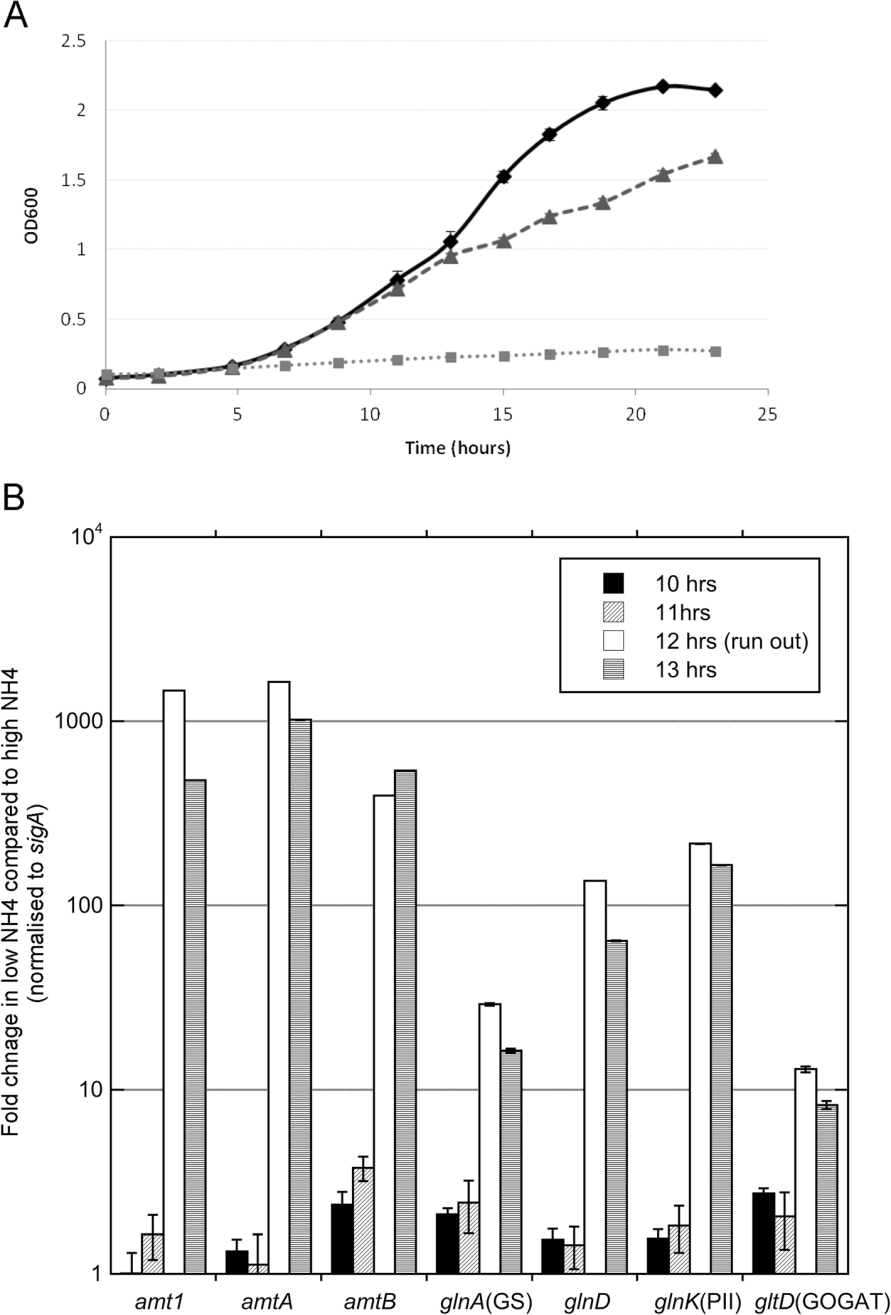
**The transcriptional response and growth of *M. smegmatis *under different nitrogen conditions. (A)** Growth of *M. smegmatis* in 0 mM (closed squares), 1 mM (closed triangles) and 30 mM (closed diamonds) ammonium sulphate (n = 3) **(B)** Average fold change in transcript levels for *amt1*, *amtA, amtB*, *glnD, glnK, gltD* and *glnA1* during nitrogen limitation compared to nitrogen excess (n = 3). Expression data was normalised to *sigA*. Error bars represent SD. Significant induction of nitrogen stress response is observed at 12 hours.

Optimisation of the nitrogen stress conditions used in this study has been described [22]. Briefly, *M. smegmatis* was grown for 24 hours in medium containing either 1 mM ammonium sulphate (nitrogen limiting) or 30 mM ammonium sulphate (nitrogen rich). Ammonium was completely depleted from the 1 mM ammonium sulphate medium between 11 and 13 hours of growth, coincident with a reduction in bacterial growth rate (Figure 1A). To determine when the nitrogen stress response occurred, transcript levels of genes known to be induced by nitrogen limitation in *M. smegmatis*[21,22] were monitored over 8 – 16 hours. Significant induction of the ammonium transporters (*amtA*, *amtB and amt1*), GOGAT small sub-unit (*gltD*), adenyl transferase (*glnD*), glutamine synthetase (*glnA1*), and PII (*glnK*) transcript levels were observed once ammonium levels in the external medium were completely depleted after 12 hours (Figure 1B).

### Expression profiling of the nitrogen stress response

**Figure 2:**
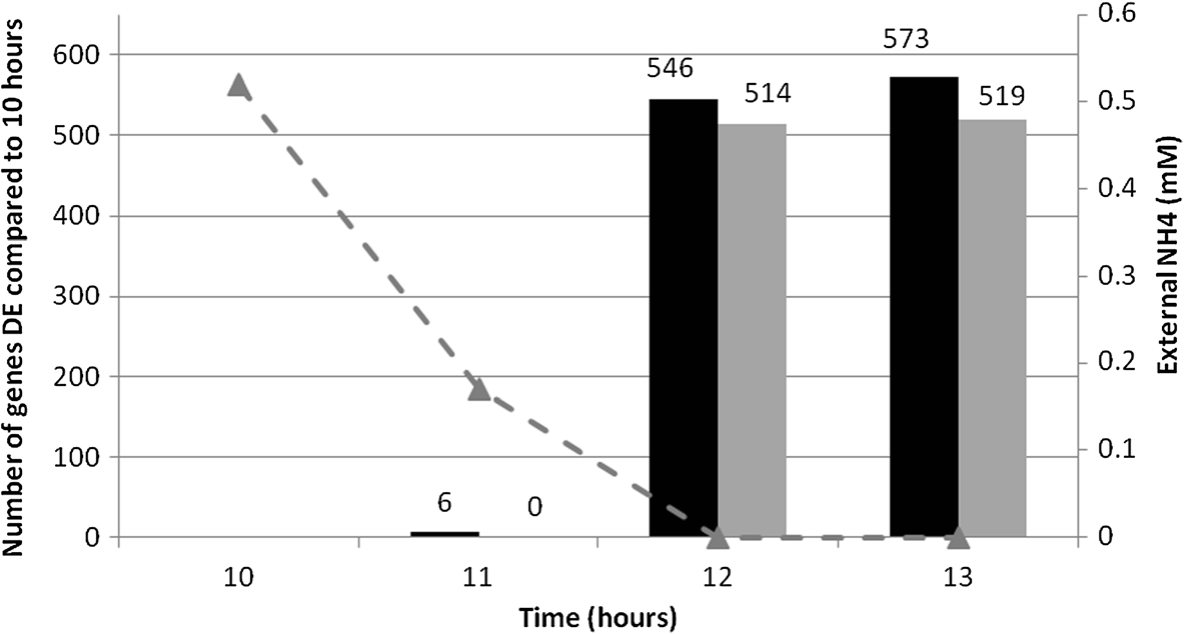
**Changes in the mycobacterial transcriptome in response to nitrogen stress.** Significant gene expression response is observed upon external nitrogen depletion. Very few genes display changes in expression until external depletion, where approximately 1000 genes are differentially expressed in nitrogen limiting and not nitrogen excess environments. Up-regulated genes are represented by black bars and down regulated genes represented by grey bars. Microarray expression data was obtained from three independent biological replicates. The concentration of ammonium (mM) in the external medium is represented by a grey dashed line with closed triangles.

RNA samples (three biological replicates, 1 mM nitrogen starting concentration) were taken at four time points (10, 11, 12 and 13 hours) and applied to a microarray to follow the depletion and eventual run out of nitrogen. Fully annotated data have been deposited in BμG@Sbase (accession number E-BUGS-140; http://bugs.sgul.ac.uk/E-BUGS-140) and also ArrayExpress (accession number E-BUGS-140), and can be viewed in Additional file 1. The data was analysed to determine which genes were significantly differentially expressed at each time point during nitrogen limitation (t = 11, 12, 13 hours) compared to nitrogen replete (t = 10 hours). Genes were considered to be significantly differentially expressed if their expression changed > 2-fold with a false discovery rate (FDR) corrected P-value <0.01, compared to their expression at 10 hours. A complete list of differentially expressed genes identified by these criteria can be viewed in Additional file 2. Consistent with the qRT-PCR analysis, there was no significant changes in genes expression until the nitrogen run out; only six genes were differentially expressed (all up-regulated) one hour before nitrogen run out (11 hours). At nitrogen run out over 1000 genes (approximately 16% of the genome) changed expression levels by greater than 2-fold: 547 genes up-regulated and 510 genes down-regulated (12 hours), with 574 genes up-regulated and 516 genes down-regulated at 13 hours (Figure 2; Additional file 2). Although it is convenient to apply a 2-fold cut off for comparison to other published stress responses, the application of an arbitrary cut off can mask less dramatic changes in gene expression and metabolism that may contribute to a coordinated response at the level of the metabolic pathway. Therefore, the AMBIENT and KEGG analyses used in this study compared the expression levels of all 6758 genes at 12 hours, during nitrogen stress, compared to nitrogen-replete conditions at 10 hours.

### Metabolic analysis of the nitrogen stress response

A draft-quality metabolic network for *M. smegmatis* was generated using the Model SEED, a webserver for the computerized generation of genome-scale metabolic models [29,31]. Regions of this network that were affected by the transcriptional response to nitrogen limitation were identified using AMBIENT [30]. These regions correspond to connected metabolic subnetworks that are coherently upregulated or repressed by the measured changes in gene expression and are unbiased by *a priori* pathway definitions. AMBIENT identified 20 candidate up- and down-regulated active modules in the metabolic network, involving a total of 330 enzyme-encoding genes (out of 963 genes included in the model) that showed a co-ordinated response to nitrogen limitation (Table 1). Of the 40 modules found by AMBIENT, all of the up-regulated modules were significant with a q-value (p-value corrected for multiple testing) of < 0.05 and six down-regulated modules were significant.

**Table 1:** AMBIENT modules differentially regulated in response to nitrogen stress

**Module**	**q-value**	**No. of reactions**	**No. of metabolites**	**Reactions with associated genes**	**Specific function(s)**
Up					
u1	<0.004	8	2	8	Nitrate/nitrite uptake and assimilation
u2	0.00	3	2	3	Cytosine/uracil uptake and assimilation
u3	0.02	6	2	6	Ethanolamine uptake and assimilation
u4	0.02	4	2	4	Arginine/ornithine metabolism
u5	0.02	4	3	4	Histidine degradation
u6	0.01	1	1	1	Arginine metabolism
u7	0.02	1	1	1	Ammonium transport
u8	0.02	4	1	4	Cation transport
u9	0.02	1	1	1	Cyanate degradation
u10	0.02	5	4	5	Fatty acid metabolism
u11	0.02	1	1	1	Hydrogen peroxide metabolism
u12	0.02	1	1	1	Hydrogen peroxide metabolism
u13	0.02	1	1	1	Hydrogen peroxide metabolism
u14	0.03	3	2	3	Energy metabolism
u15	0.03	2	1	2	Thiamine transport and assimilation
u16	0.04	4	1	4	Galactose metabolism
u17	0.03	1	1	1	Allantoin transport
u18	0.03	1	1	1	Hydrogen peroxide metabolism
u19	0.04	3	1	3	Gluconate degradation
u20	0.04	2	1	1	Fatty acid metabolism
uHP	<0.004	4	1	4	Hydrogen peroxide metabolism
Down					
d1	<.004	152	75	110	LPS, RNA and DNA synthesis, central carbon metabolism
d3	0.04	5	3	5	Pantothenate metabolism
d4	0.04	4	2	3	Fatty acid metabolism
d5	0.04	4	3	0	Isoprenoid utilization
d6	0.05	4	2	3	Fatty acid metabolism
d7	0.04	3	2	2	Xylose uptake and assimilation

Up- and down-regulated modules found using AMBIENT in nitrogen stress, in decreasing order of score, indicating the number of reactions and metabolites present in each. Scores are calculated by AMBIENT, taking into account the logarithm of the fold-change of the genes associated with each reaction and the connectivity in the metabolic network of each metabolite in the module. The number of genes associated with these reactions according to the Model SEED *M. smegmatis* model is also shown. Functional assignments are based on manual inspection of the exact function of the member reactions of the modules. Diagrams for each module are available as Additional files 3 (Up modules) and 4 (down modules), except for u11, u12, u13 and u18, which are combined in uHP.

AMBIENT analysis of metabolism is system wide and as therefore attempts to take into account reactions for which there are no data. Due in part to the draft nature of the metabolic model used, and in part to the more general problem of known reactions without known associated enzymes, many reactions could not be assigned scores from the data available. For all reactions without an assigned score, AMBIENT assigned the median reaction score. This approach has been found to be very useful in finding coordinated metabolic changes using AMBIENT, but occasionally it produces modules in which none of the reactions have associated genes with expression change data. In this case, where all of the reactions have been assigned the default median expression score value there is no experimental evidence indicating how that module has responded to the environmental change of interest. AMBIENT found one such down-regulated module that was excluded from further analysis.

The q-value, number of reactions, number of genes and areas of metabolism identified during nitrogen stress are summarised in Table 1. All significantly changed network module diagrams, along with a list of genes in each module, can be viewed in Additional files 3, 4 and 5.

### Reaction directionality

In this work, several areas of metabolism have been highlighted as being significantly affected by transcriptional changes in response to nitrogen starvation. However, due to the unavailability of a high quality curated metabolic model for *M. smegmatis* it was not possible to apply FBA-based analysis (such as Differential Producibility Analysis [26] or E-Flux [27]) to this bacterium, flux directions or direction changes for any reaction in the metabolic network are difficult to predict. Hence here we make no definitive claims about flux changes, except where their directions are obvious from the identity or context of the relevant reaction.

### Up-regulated active modules

Although a general decrease in metabolic activity is expected due to the observed reduction in growth rate upon nitrogen limitation (Figure 1A), there were many significant increases in metabolic activity, which are likely to be directly related to nitrogen availability. It can be seen from Table 1 that twenty active modules involved in distinct metabolic functions were up-regulated when the cells are exposed to nitrogen stress, with another six, the majority of which are associated with the reduced growth rate, down-regulated.

#### Nitrogen metabolism: uptake, assimilation and scavenging

AMBIENT analysis showed that 8 of the top 9 modules significantly induced in nitrogen stress are involved in nitrogen uptake, scavenging and assimilation, highlighting these reactions as important survival mechanisms. Soil is an extremely rich source of inorganic and organic nitrogen and it appears that when nitrogen is limited, *M. smegmatis* induces a variety of scavenging pathways to utilise other available nitrogen sources in the environment and compete for resources. To assess how the modules identified are interrelated, modules were extended to include all metabolites connected to each member reaction of those modules. The resulting network was plotted and it was seen that many modules were linked by various metabolites. Additional file 6 shows the largest connected component of this subnetwork. In S6.2 modules members are coloured the same and it can be seen that many of the modules found are closely associated, where substrates (or products) of reactions in one module are substrates (or products) of reactions in another, especially around ammonium. All of the uptake and scavenging pathways induced produce ammonium as the final breakdown product, which is subsequently assimilated into cellular biosynthetic donors.

**Figure 3:**
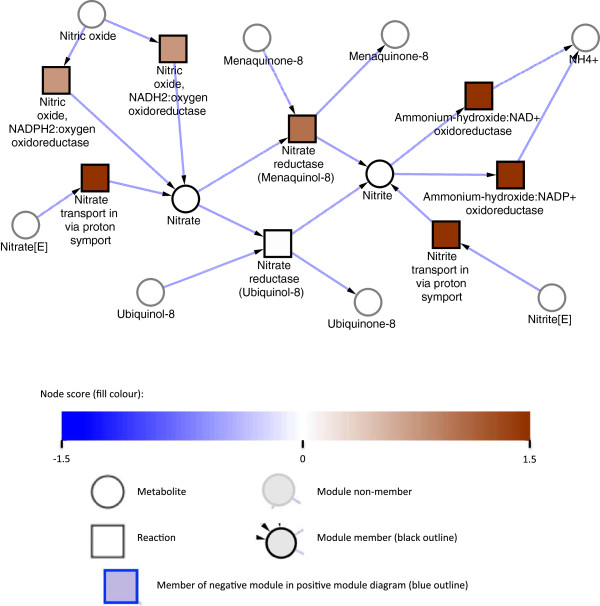
**Assimilation of nitrate and nitrite in response to nitrogen stress.** Metabolic module u1 representing nitrate/nitrite assimilation consisting of 8 reactions and 2 metabolites with a score of 13.9 and a q-value of <0.004. Squares represent enzymatic reactions and circles represent metabolites. No definitive claims can be made about fluxes, so arrows are only indicative and taken from the identity or context of the relevant reaction.

Nitrate/nitrite uptake and assimilation (u1) is the metabolic module most significantly up-regulated in nitrogen stress (Figure 3; Table 1). Nitrates and nitrites are available in the soil as they are major constituents of organic waste material, and two nitrate/nitrite transporters (MSMEG0433 and MSMEG5141) are up-regulated upon nitrogen limitation. Once inside the cell, nitrate is converted into nitrite by nitrate reductase (MSMEG2837 (NarB), NarH, NarI, NarJ, MSMEG5140 (alpha-subunit)), then further broken down by nitrite reductase (NirB, MSMEG0428; large and small subunits) into ammonium. Both reductase enzymes require the presence of an electron acceptor (ubiquinol or menaquinol) for activity, with the menaquinol reaction up-regulated; preferential use of menaquinol in *E. coli* is associated with anaerobic growth [32], and may be a result of the decreased growth rate seen here. Interestingly, MSMEG1336, is also included in this network module; the predicted reaction of this enzyme is the conversion of nitric oxide into nitrate, identifying another potential source of environmental nitrogen.

Pyrimidines, purines, and ethanolamine are present in soil, originating from other microbes. Cytosine and uracil uptake pathways, and deaminases to breakdown cytosine to uracil and ammonium (module u2) are up-regulated, as is ethanolamine utilization (module u3). The fate of the uracil is unclear, since the module utilising it is down regulated (d1). However the enzymatic conversion of uracil into barbituric acid, which can then be metabolised into urea and malonic acid, has been described for mycobacteria [33].

Metabolism of Arginine/Ornithine (u4, u6), Histidine (u5), ammonium transport (u7), and thiamine transport (u15) are all shown in Additional file 3. Ammonium is the preferred nitrogen source in most bacteria and *M. smegmatis* contains 3 ammonium transporters, suggesting that it is also the preferred nitrogen source in this species. Amt1 (MSMEG6259) was up-regulated 133-fold, AmtA (MSMEG4635) was up-regulated 144–fold and AmtB (MSMEG2425) was up-regulated 16-fold. AmtB shows basal levels of transcription when nitrogen is present, whereas the other 2 transporters are only transcribed during stress. MSMEG4635 is missing from Model SEED, but the ammonium transport module (u7) contains the others genes.

The ability to continue growing in the apparent absence of nitrogen (albeit at reduced growth rate) *in vitro* is an interesting observation. A huge environmental nitrogen source scavenging response is initiated, but nitrogen sources such as urea, nitrate, and ethanolamine are absent from the growth medium. Metabolically labelling the nitrogen source and monitoring its utilisation could be used to investigate the pathways. It is possible that mycobacteria break down internal stores of nitrogen, such as DNA, protein or cell wall components. This reduction in growth rate may be a general stress response or a pre-requisite to allow the re-allocation of cellular components into nitrogen metabolism. Investigations to study this are in progress.

#### Hydrogen peroxide metabolism

**Figure 4:**
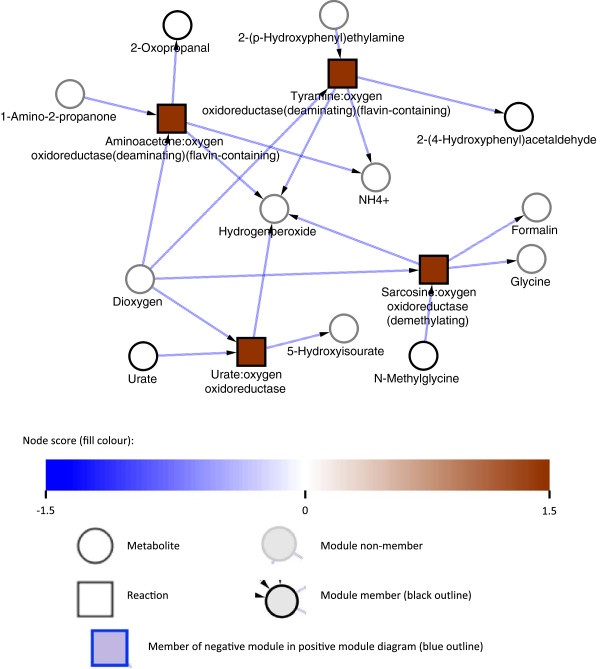
**Regulation of hydrogen peroxide metabolism in response to nitrogen stress.** Metabolic modules uHP representing hydrogen peroxide metabolism consisting of 6 reactions and 1 metabolite with a combined score of 9.77 and a q-value < 1e-5. Squares represent enzymatic reactions and circles represent metabolites. No definitive claims can be made about fluxes, so arrows are only indicative and taken from the identity or context of the relevant reaction.

Four small modules (u11, u12, u13 and u18) linked to Hydrogen peroxide metabolism show up in the top 20 modules list (Table 1), each containing one reaction and one metabolite. These were combined into the single module uHP (Figure 4) with hydrogen peroxide to connect them, and scored by AMBIENT, at 9.77 (q-value < 1e-5), which would put it second (by score) in the list of up-regulated modules (Table 1). AMBIENT did not initially pick up this module because hydrogen peroxide is highly connected as the substrate/product of 12 different reactions in the metabolic network, and is therefore penalised by the AMBIENT scoring system. Up-regulation of peroxide metabolism is a surprising finding, suggesting that pathways normally associated with oxidative stress protection are activated in nitrogen stress. Detoxification may form part of a general response to stress with the cell priming itself for survival in harsh environments. However, the most highly up-regulated gene in nitrogen stress (MSMEG2526, an amine oxidase) is up-regulated over 500 fold, and releases hydrogen peroxide during the breakdown of primary amines into ammonia. If transcript levels translate into enzyme activity then this could explain the observed oxidative stress response identified by AMBIENT, as the cell has to detoxify the ROS. The metabolic model generated by Model SEED does not contain the reactions catalysed by *ahpC* and *ahpD*, which are therefore absent from the module although they are both up-regulated at least 2 fold (Additional file 2) and so could protect the cell from the toxic effects of hydrogen peroxide produced in this metabolic reaction. This highlights a limitation of AMBIENT that can be overcome by studying individual gene expression data directly where gene function is known but the annotation is lacking in the metabolic model generated by Model SEED.

#### Other modules

Another module induced in nitrogen stress is involved in cation transport (module u8), with Cobalt/Zinc/Cadmium resistance protein (MSMEG0755). Potassium transport in module u8 involves the reaction catalysed by MSMEG2769, a putative TrkB protein. This protein is involved in membrane potential homeostasis and antibiotic resistance [34]. This system may prime the bacteria for stress survival by ensuring membrane homeostasis is maintained, or it could be required for the large scavenging response and increased membrane transport observed. Inactivation of this gene led to reduced osmotic stress survival [34], but the ability of this mutant to survive nitrogen stress is unknown.

The remaining up-regulated modules are involved in processes induced when *M. smegmatis* growth rate was reduced in conditions of low energy and hypoxia [16]; adaptation to low growth rate induced the expression of alternative primary dehydrogenases and hydrogenases for energy generation and unique pathways for scavenging carbon. We also observed the up-regulation of a number of modules involved in carbon metabolism/degradation (u14, u16, and u19), and fatty acid metabolism (u10 and u20), which may also be a consequence of the reduced growth rate observed under nitrogen limitation. Module u9 highlights a limitation in the genome annotations in the Model SEED generated metabolic model, incorrectly annotating the gene as a cyanate hydrolyase, converting cyanate to amino-formic acid, whereas the alternative annotation in other databases is a cyanate hydratase catalysing the degradation of cyanate to ammonia and carbamate. This second annotation would seem to make more biological sense.

### Down-regulated active modules

The decrease in growth rate imposed by nitrogen stress has a general impact on metabolism that is reflected in the large number of reactions (405) associated with down-regulated genes. It is worth noting that those modules found to be significantly down-regulated at the 5% level show a coordinated response beyond that expected by random chance, implying that they are relevant to the way in which *M. smegmatis* balances its metabolism in response to a single stress. Module d1 is the largest metabolic module significantly affected by nitrogen limitation that was identified by AMBIENT. It includes parts of metabolism directly related to growth, such as LPS biosynthesis (A), central carbon metabolism (B), RNA biosynthesis (C), and DNA biosynthesis (D), with a total of 129 reactions and 61 metabolites (Additional file 4). In addition to the metabolites identified in the module, these reactions link to many other metabolites throughout the metabolic network, totalling between them 191 metabolites (17% of the total metabolites in the entire *M. smegmatis* metabolic network). However, protein synthesis and amino acid biosynthesis do not appear to be affected by the growth change, or at least they are not transcriptionally controlled under these conditions.

Module d1 illustrates the capability of AMBIENT to identify modules that contain metabolic reactions for which there is no gene annotation information. There are 9 reactions in d1 that do not have gene assignments in the metabolic model used in this work. These include the glucosyl transferase reactions converting UDP-alpha-D-glucose to UDP as well as the ‘DNA synthesis’ reaction, which represents the conversion of nucleic acids into the DNA molecule as part of normal bacterial growth. Two modules related to fatty acid metabolism are down-regulated (d4 and d6), while two (u10 and u20) are up-regulated, suggesting there may be a redistribution of resources as a consequence of the reduced growth rate and nitrogen stress. The apparently very highly connected negative metabolic response to nitrogen starvation, as opposed to the more fragmented up-regulated response, is probably due to the large number of down-regulated reactions, rather than representing a more coherent down-regulated response.

### Comparison of modules identified by AMBIENT with those found by KEGG pathway enrichment

A variety of methods have been used to assess the enrichment of pre-defined sets of genes with respect to differential expression. As a comparison to AMBIENT, we applied Fisher’s exact test to study the enrichment of KEGG pathways for up- and down-regulated genes (Table 2). Several pathways were found to be down-regulated, but only two pathways (msm00260 and msm00350, both involved in amino acid metabolism) were significantly up-regulated, highlighting the potential limitations of this type of analysis. The nitrogen metabolism KEGG pathway was not identified as significantly induced at a false discovery rate of 5%. Up-regulated amino acid metabolism may indicate that the stressed cells are altering their amino acid usage, or scavenging nitrogen from cellular sources. Down-regulated KEGG pathways are presumed to be a general response to the reduced growth rate, with some pathways (lipid and carbohydrate metabolism) overlapping with the AMBIENT analysis.

**Table 2:** KEGG pathways identified using genes differentially expressed under nitrogen stress

**KEGG pathway**	**KEGG ID**	Size*	**p-value**	**q-value**
Up				
Glycine, serine and threonine metabolism	msm00260	41	0.0002	0.0243
Tyrosine metabolism	msm00350	41	0.0003	0.0243
Down				
Carotenoid biosynthesis	msm00906	3	0.0000	0.0000
Geraniol degradation	msm00281	72	0.0002	0.0025
Benzoate degradation	msm00362	94	0.0002	0.0025
Vitamin B6 metabolism	msm00750	6	0.0006	0.0069
Propanoate metabolism	msm00640	94	0.0015	0.0162
Peptidoglycan biosynthesis	msm00550	16	0.0022	0.0223
Valine, leucine and isoleucine degradation	msm00280	91	0.0025	0.0238
One carbon pool by folate	msm00670	14	0.0031	0.0247
Fatty acid metabolism	msm00071	94	0.0032	0.0247
Terpenoid backbone biosynthesis	msm00900	23	0.0040	0.0259
Phenylalanine, tyrosine and tryptophan biosynthesis	msm00400	27	0.0087	0.0470

KEGG enrichment analysis on all DE genes at 10 vs 12 hours. Pathways shown are significant under a one-tailed Fisher’s exact test at a FDR of 5%. *Size denotes number of genes on the microarray mapped to that KEGG pathway.

In this study, the standard enrichment analysis failed to give an adequately specific and flexible picture of general metabolic activity. Its reliance on pre-defined pathways (or gene sets) means that it operates at a more coarse-grained level of metabolism, which risks missing species-specific pathways, cross-pathway modules and small modules comprising only a few reactions. AMBIENT addresses this problem by allowing modules to form between any connected reactions, not just those within a predefined set of reactions. Additionally, AMBIENT takes advantage of the information on metabolite connectivity available in the metabolic network, favouring metabolites with lower connectivity as pathway members but also allowing those with higher connectivity where it is justified by the data. Further, AMBIENT can take into account reactions with unmeasured genes and gene measurement errors to provide a robust approach to finding specific areas of metabolism affected by particular environmental changes. In this study, an additional benefit of AMBIENT over KEGG is that the metabolic model used includes many additional genes and gene-reaction relationships. KEGG gives an overview of the changes occurring due to Nitrogen run-out but cannot (by its nature) give specific details of the sets of adjacent reactions affected in this environment. By looking at this finer level AMBIENT has shown co-ordinated up-regulation of reactions involved in Nitrogen scavenging and metabolism – missed in the coarser grained KEGG pathway analysis. The most notable example of this is in Module u3, ethanolamine transport and assimilation, in which 6 genes out of 7 are not present in KEGG. There are also several genes identified by the active modules approach that are not annotated in the *M. smegmatis* genome (i.e. do not have MSMEG numbers) such as *Gamma-glutamyl phosphate reductase* (EC 1.2.1.41) in u5 and *Aconitate hydratase (EC 4*.*2*.*1*.*3*) and *2-methylisocitrate dehydratase* (EC 4.2.1.99) in u15, or that have different annotations compared to Smegmalist, for example MSMEG1414 is probably more correctly annotated as an amidinotransferase, rather than a dimethylarginase. These discrepancies are probably due to an out of date annotation on the NCBI website from which the MSMEG numbers are acquired, combined with Model SEED’s own annotation using the RAST server. AMBIENT has some drawbacks, such as finding modules without any experimental evidence to support them, but these are trivial to identify and exclude from further analysis. Overall the benefits of AMBIENT far out-weigh the limitations, with organism-specific databases and BioCyc used to supplement Model SEED where appropriate.

## Conclusions

The ability to sense and respond to changing environments is crucial for the survival of bacteria, and modulation of gene expression is central to this adaptation; however, to date a study of the global gene expression response to nitrogen limitation in mycobacteria has not been performed. In order to address this shortcoming, we studied the global genetic response to nitrogen stress in *M. smegmatis* by combining global transcriptional profiling with a network-based approach to search for patterns in gene transcriptional changes in the metabolic network. These combined approaches identified over 1000 genes and several areas of metabolism that changed significantly in response to nitrogen stress. This included reduced growth rate, lower energy demand in the cell, reduced metabolic rates, carbon catabolism and biosynthesis of RNA, protein and lipid, with increased nitrogen uptake and assimilation. Growth rate reduction in conjunction with scavenging nitrogen from other sources could represent an important mechanism by which mycobacteria survive nutrient limitation until more favourable conditions arise. The AMBIENT approach provided much more detailed insights than the standard KEGG enrichment analysis, proving to be a valuable tool to translate gene expression changes into metabolic responses in the absence of a high quality curated metabolic model, and producing meaningful insights into mycobacterial nitrogen stress survival strategies.

## Methods

### Bacterial culture and nitrogen limiting conditions

Cultures were taken from a frozen seed stock of *M. smegmatis* mc^2^155 (ATCC 700084). Cells were thawed and grown to late log phase at 37°C with shaking at 180 rpm in Sauton’s minimal medium [35] supplemented with 0.2% glycerol, 0.015% Tyloxapol and 0.005% zinc sulphate. Cells were washed twice in nitrogen free Sauton’s minimal medium and diluted to a starting OD_600_ of 0.08 in nitrogen free Sauton’s containing either 1 mM or 30 mM ultra-pure ammonium sulphate (Sigma). Sauton’s nitrogen-free minimal medium was as described above except it contained ferric citrate instead of ferric ammonium citrate and the asparagine was removed. Cells were grown over 24 hours with growth monitored by OD_600_ and cfu ml^-1^.

### RNA isolation

*M. smegmatis* was cultures under nitrogen limitation (1 mM) or nitrogen rich (30 mM) conditions. Three independent cultures for each condition were sampled during the period nitrogen is depleted from the 1 mM culture (10–13 hours growth). Briefly, cells were immediately added to 5 M GTC and harvested by centrifugation. Pellets were resuspended in TRIzol (Life Technologies) and stored at −80°C. The thawed cell/trizol suspensions were transferred to tubes containing Zirconium beads (MP Biomedicals) and the cells lysed in a ribolyser Fastprep (Hybaid) for two cycles of 30 seconds on the maximum setting. Samples were then centrifuged and the TRIzol supernatants added to chloroform. After a second round of chloroform extraction, the RNA/DNA was then precipitated with isopropanol and the resulting nucleic acids resuspended in RNA secure (ABI Life Technologies). The RNA was then purified using the RNeasy kit (Qiagen) according to manufacturer’s instructions except two rounds of DNAse treatment were performed: one on-column digest **(Invitrogen)** prior to elution of the RNA from the column and one Turbo DNase treatment (Ambion) on the eluted RNA. Superase (ABI Life Technologies) was added to the RNA to protect it from degradation and stored at −20°C. RNA quality and quantity was determined by OD 260/280 and 260/230, gel electrophoresis and bio-analyzer analysis.

### Quantitative real-time PCR (qRT-PCR)

To determine gene expression levels, cDNA was amplified from 100 ng of RNA using the SuperScript III First-Strand Synthesis SuperMix **(Invitrogen)**. qRT-PCR reactions were carried out in a final volume of 10 μl (1 μl of cDNA, 5 μl of TaqMan PCR master mix (Applied Biosystems), 0.5 μl of the appropriate TaqMan probe (Applied Biosystems)). Amplification was performed on an Applied Biosystems 7500 Real-Time System (conditions 50°C 5 min, 95°C 10 min, and 40 cycles of 95°C 15 sec, 60°C 1 min). Linear amplification and amplification efficiencies for each TaqMan primer/probe set was determined. Real-time analysis was performed on RNA from three independent cultures and quantification of *sigA* expression served as an internal control. Fold changed were calculated as a ratio of the arbitrary expression units, standardised to *sigA*, between the nitrogen excess and limiting conditions. Statistical analysis of data was performed using a Student’s *t*-test, a *P* value of ≤ 0.01 was considered significant. Primers and Taqman probe sequences for each gene studied are given in Additional file 7.

### Preparation of labelled cDNA from total RNA

Labelled cDNA was prepared from 1.5 μg total RNA using Cy3-dCTP (GE Healthcare) and SuperScript II reverse transcriptase with random hexamer primers (Life Technologies – **Invitrogen**). Agilent One Color Spike-In controls were labelled together with the RNA samples according to manufacturer’s instructions. Labelled cDNA was purified by Qiagen MinElute column, combined with 10× CGH blocking agent and 2× Hi-RPM hybridisation buffer (Agilent) and heated at 95°C for 5 minutes prior to loading onto microarray slides which were incubated overnight in an Agilent rotating oven at 65°C, 20 rpm. After hybridization, slides were washed for 5 minutes at room temperature with CGH Wash Buffer 1 (Agilent) and 1 minute at 37°C with CGH Wash buffer 2 (Agilent) and scanned immediately, using an Agilent High Resolution Microarray Scanner, at 2 μm resolution, 100% PMT. Scanned images were quantified using Feature Extraction software v 10.7.3.1.

### Microarray design

The microarray was constructed by determining all unique genes from the 6887 chromosomal predicted coding sequences of *M. smegmatis* strain MC2 155, downloaded from Ensembl Bacteria Release 5 (http://bacteria.ensembl.org/). Multiple optimal hybridisation 60-mer oligonucleotide sequences were designed (Oxford Gene Technologies), from which a minimal non-redundant subset of oligonucleotides were selected with target coverage of three 60-mers per gene. Arrays were manufactured on the Inkjet in-situ synthesized platform (Agilent) using the 8x60k format. The full array design is available in BμG@Sbase (BμG@Sbase: A-BUGS-40) and also in ArrayExpress (ArrayExpress: A-BUGS-40). Fully annotated microarray data have been deposited in BμG@Sbase (accession number [A-BUGS-140]) and also ArrayExpress (accession number [A-BUGS-140]).

### Statistical analyses of differential gene expression

Statistical analyses of the gene expression data was carried out using the statistical analysis software environment R together with packages available as part of the Bioconductor project (http://www.bioconductor.org). Data generated from the Agilent Feature Extraction software for each sample was imported into R. Replicate probes were mean summarised and quantile normalised using the preprocess Core R package. The limma R package [36] was used to compute empirical Bayes moderated *t*-statistics to identify differentially expressed gene between time points. Generated p-values were corrected for multiple testing using the Benjamini and Hochberg False Discovery Rate. A corrected p-value cut-off of less than 0.01 was used to determine significant differential expression.

### KEGG enrichment analysis

Metabolic pathway enrichment for strongly up- and down-regulated genes was assessed using the one-tailed Fisher's exact test against the KEGG pathway assignments for *M. smegmatis*. Correction for multiple testing was performed using the q-value function in the Bioconductor R package to estimate false discovery rates (FDRs) [37]. Pathways were assigned as significantly enriched if they had a FDR below 5%.

### AMBIENT (Active Modules for BIpartitE NeTworks) analysis

A modified active modules approach [38] called AMBIENT [30] was applied to the transcriptomic data to identify, in an unbiased way, areas of the metabolic network that are notably affected when *M. smegmatis* is exposed to nitrogen stress. The active modules approach used in AMBIENT takes into account the bipartite nature of the metabolic network of reactions and metabolites to find metabolic pathways (or modules) that are co-ordinately affected at the transcriptional level due to some environmental change. Co-ordinately affected modules (connected components of the metabolic network) were found containing reactions associated with large changes in gene expression, linked by metabolites with low connectivity (i.e. linked to few other reactions).

In brief, the AMBIENT method works as follows (for further details see [30]:

i. A bipartite network containing reaction nodes linked via metabolite nodes is used

ii. Scores are assigned to each reaction (calculated as the mean log fold-change of genes associated with that reaction)

iii. Weights (negative scores) are assigned to metabolites proportional to their connectivity in the metabolic network

iv. Simulated annealing is used to find the connected components of the network with the highest scores (calculated as the sum of the scores of all members of the connected component

v. The significance of the modules found is assessed empirically by looking at the scores of random sets of reactions and metabolites of the same size as the modules found

Spuriously routes via highly connected currency metabolites (e.g. water, carbon dioxide or ATP) are minimised by the assignment of connectivity-dependent weights to their nodes in the bipartite network.

## Abbreviations

AMBIENT: Active Modules for BIpartitE NeTworks; FDR: False discovery rate; GSEA: Gene set enrichment analysis; KEGG: Kyoto encyclopedia of genes and genomes; TB: Tuberculosis; GDH: Glutamate dehydrogenase; GS: Glutamine synthetase; GOGAT: Glutamate synthase; DPA: Differential producibility analysis; FBA: Flux balance analysis.

## Competing interests

The authors declare that they have no competing interests.

## Authors’ contributions

KW designed and performed experiments, analysed the data and co-wrote the manuscript. VJ performed experiments, GB generated and formatted the normalised array data and performed statistical analyses on the data, AW designed the *M. smegmatis* (v1.0.1) microarray, JP and WB performed the GSEA and AMBIENT analyses on the normalised array data and co-wrote the manuscript, and BR supervised the project and co-wrote the manuscript. All authors have approved the final manuscript for publication.

## Supplementary Material

Additional file 1 An Excel file containing the average expression ratios for all analysed genes in nitrogen limiting (Low) and nitrogen excess (High) conditions (n = 3). Click here for file

Additional file 2 An Excel file containing average fold changes (n = 3) for all significantly differentially expressed genes in nitrogen limiting conditions (greater than 2-fold change; FDR corrected p < 0.01). Click here for file

Additional file 3 A zip file containing illustrations of the 20 up-regulated metabolic network modules in nitrogen limitation identified by AMBIENT. Click here for file

Additional file 4 A zip file containing illustrations of the 6 down-regulated metabolic network modules in nitrogen limitation identified by AMBIENT. Click here for file

Additional file 5 A list of genes in each module in S3 and S4. Click here for file

Additional file 6 **Up-modules are presented in their metabolic network context, illustrating a cluster of modules around ammonium and around hydrogen peroxide.** In S6.1 the nodes in a module are coloured using the original scoring scheme, while in S6.2 the nodes in each module are coloured the same. Click here for file

Additional file 7 **A table showing the qRT-PCR primers and probe sequences used for the relative quantification of gene expression in *M. smegmatis * during nitrogen limitation.** Click here for file
