# Supplementary material for: Deciphering the response of Mycobacterium smegmatis to nitrogen stress using bipartite active modules
Source: BMC Genomics. 2013 Jul 2;14:436. doi: 10.1186/1471-2164-14-436 (PMC3706326; doi:10.1186/1471-2164-14-436)
Supplement: Additional file 3 — A zip file containing illustrations of the 20 up-regulated metabolic network modules in nitrogen limitation identified by AMBIENT. [file 1471-2164-14-436-S3.zip › add3/1851983883936012_add3.pdf]

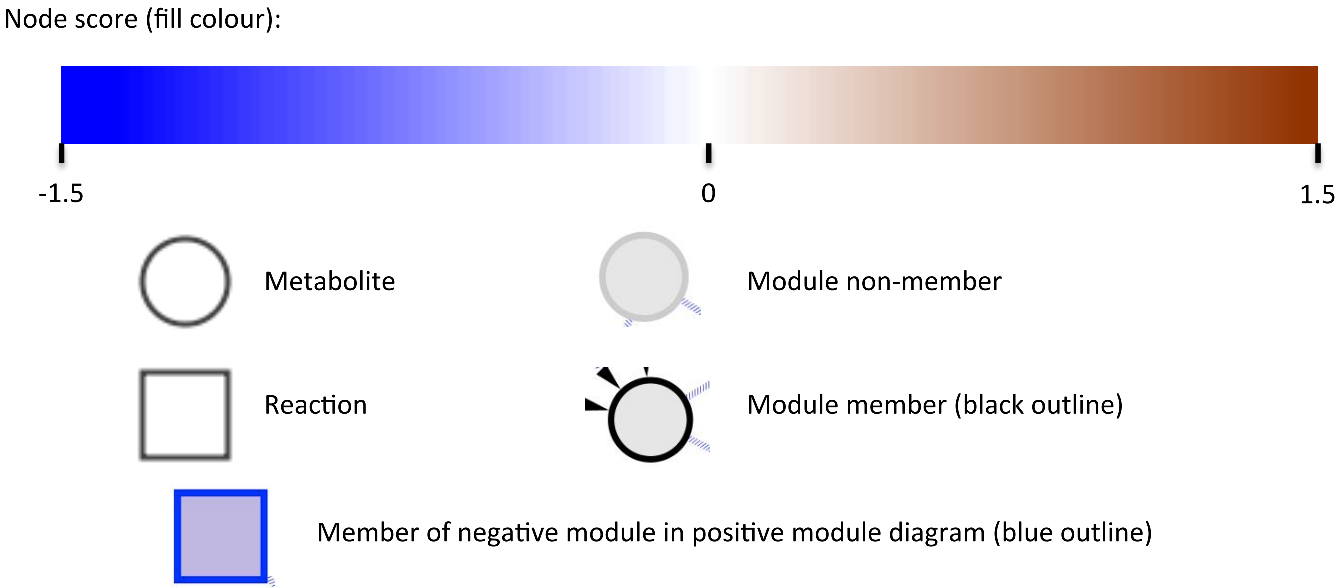
Node score (fill colour):
-1.5
0
1.5
Metabolite
Module non-member
Reaction
Module member (black outline)
Member of negative module in positive module diagram (blue outline)
