# Supplementary figures and images for: Deciphering the response of Mycobacterium smegmatis to nitrogen stress using bipartite active modules
Source: BMC Genomics. 2013 Jul 2;14:436. doi: 10.1186/1471-2164-14-436 (PMC3706326; doi:10.1186/1471-2164-14-436)

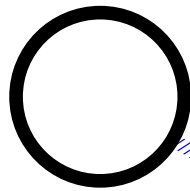

$\text{NH}_4^+[\text{E}]$

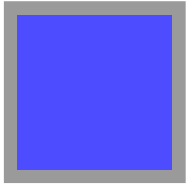

$\text{NH}_3[\text{E}]$

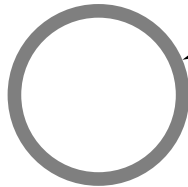

$\text{NH}_4^+$

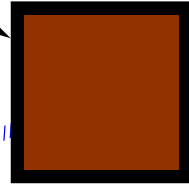

Ammonia  
transport

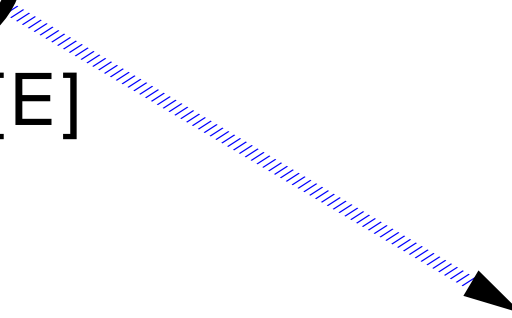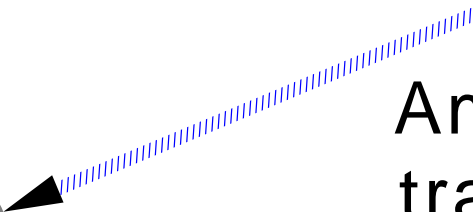

Supplement: Additional file 3 — A zip file containing illustrations of the 20 up-regulated metabolic network modules in nitrogen limitation identified by AMBIENT. [file 1471-2164-14-436-S3.zip › add3/1851983883936012_add10.pdf]

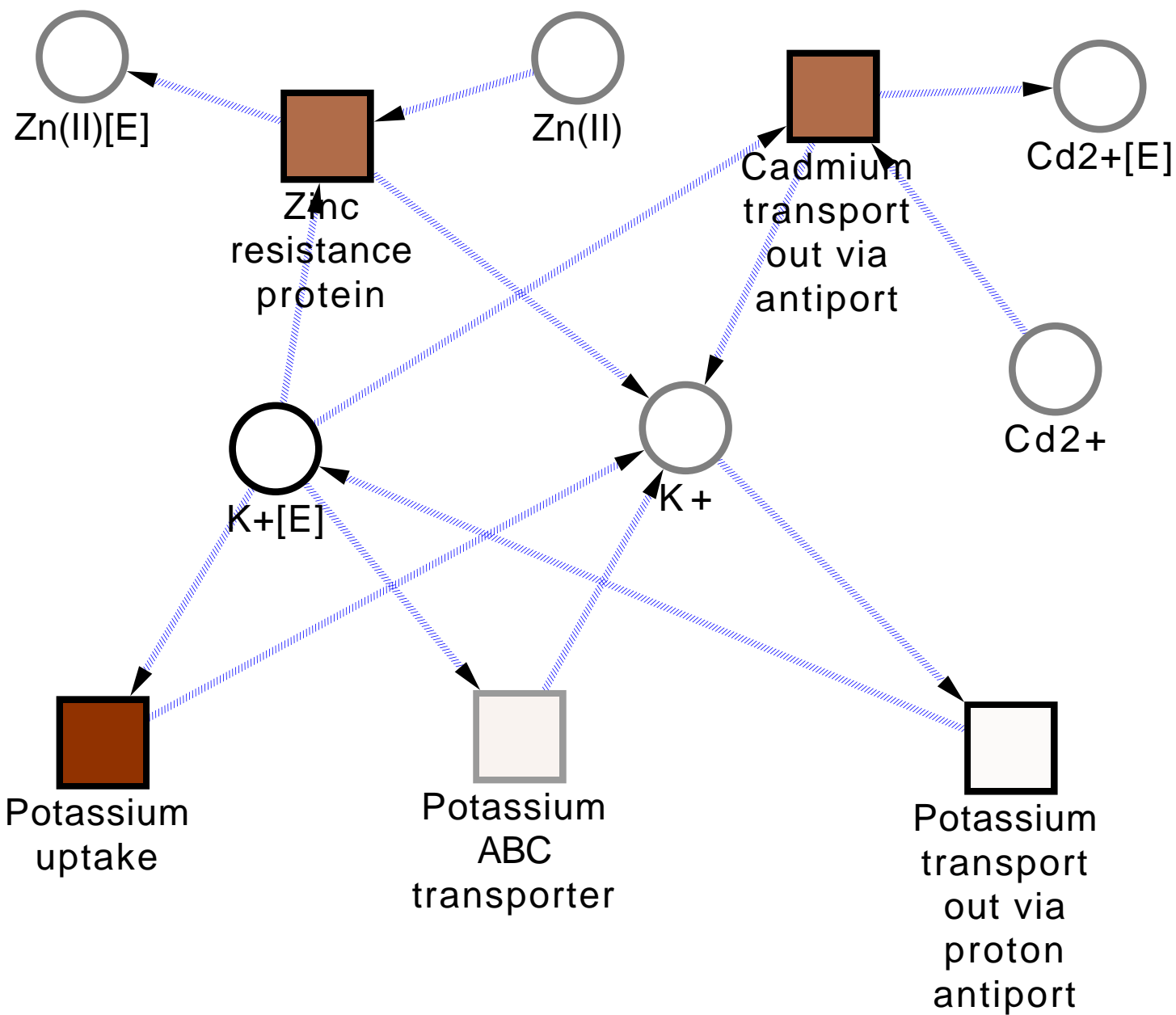

Supplement: Additional file 3 — A zip file containing illustrations of the 20 up-regulated metabolic network modules in nitrogen limitation identified by AMBIENT. [file 1471-2164-14-436-S3.zip › add3/1851983883936012_add11.pdf]

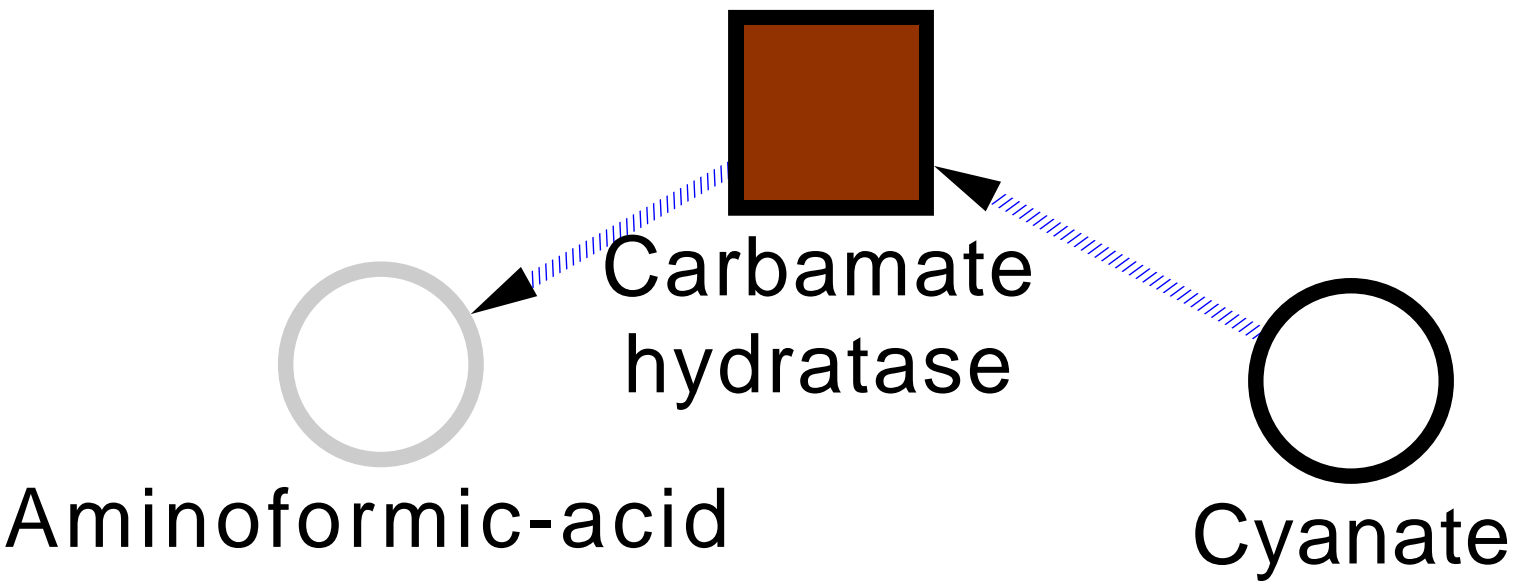

Supplement: Additional file 3 — A zip file containing illustrations of the 20 up-regulated metabolic network modules in nitrogen limitation identified by AMBIENT. [file 1471-2164-14-436-S3.zip › add3/1851983883936012_add12.pdf]

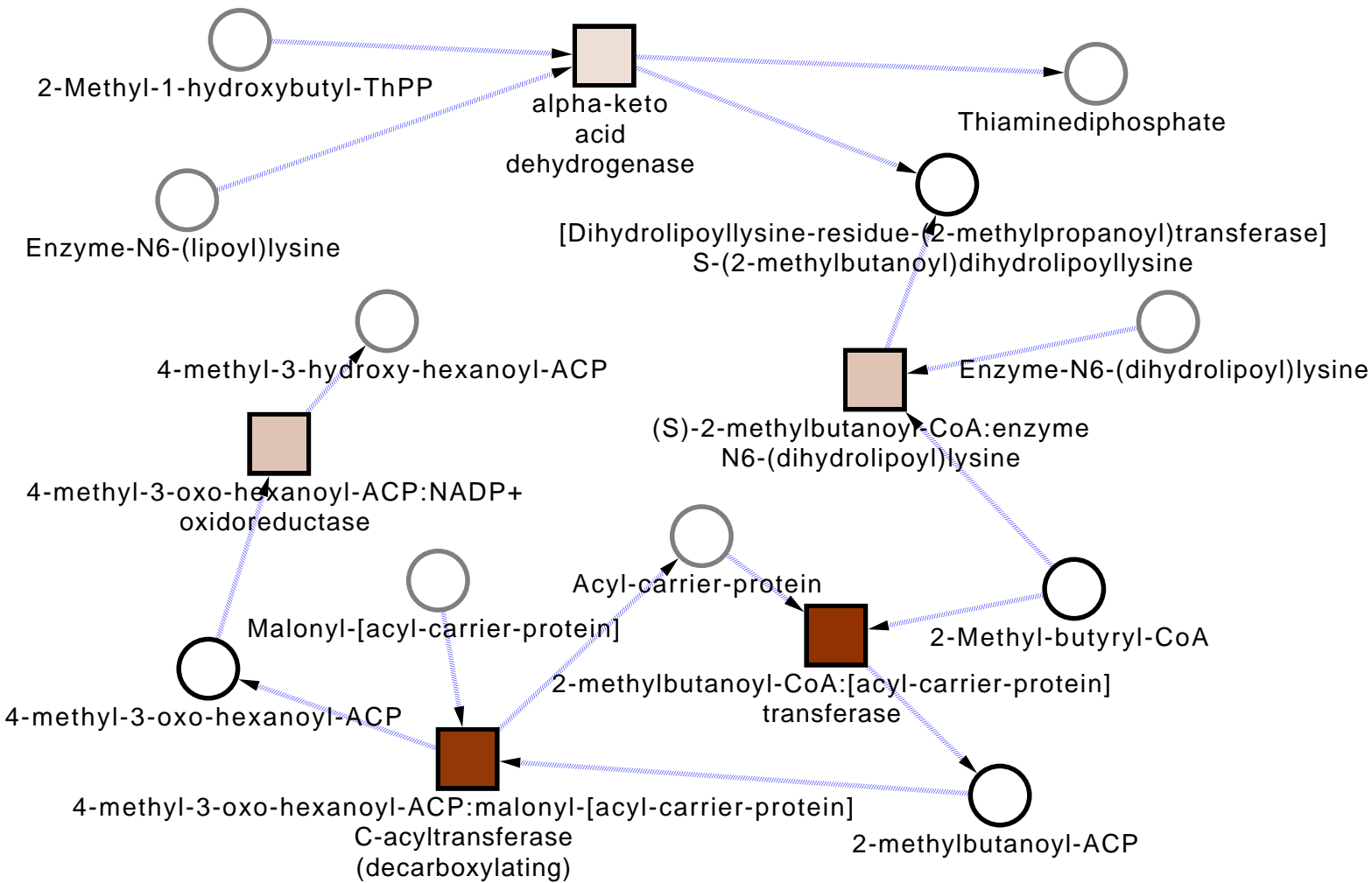

Supplement: Additional file 3 — A zip file containing illustrations of the 20 up-regulated metabolic network modules in nitrogen limitation identified by AMBIENT. [file 1471-2164-14-436-S3.zip › add3/1851983883936012_add13.pdf]

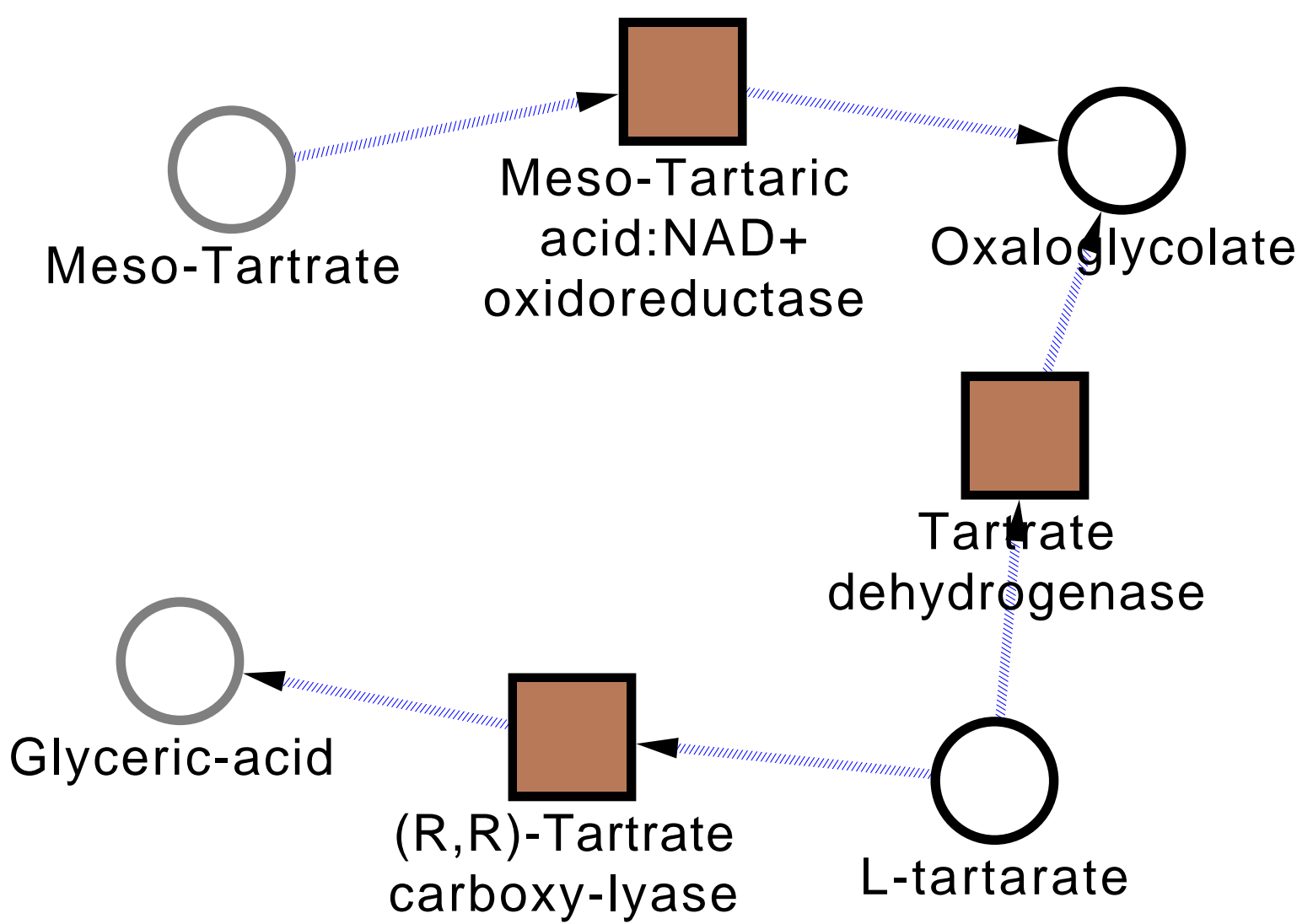

Supplement: Additional file 3 — A zip file containing illustrations of the 20 up-regulated metabolic network modules in nitrogen limitation identified by AMBIENT. [file 1471-2164-14-436-S3.zip › add3/1851983883936012_add14.pdf]

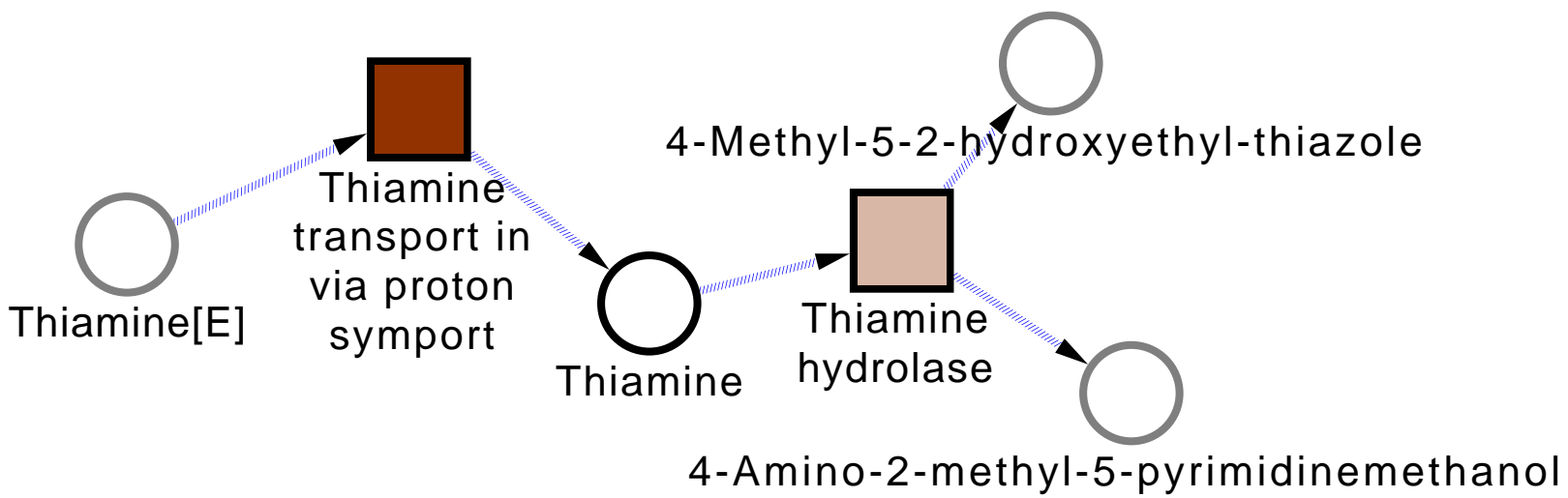

Supplement: Additional file 3 — A zip file containing illustrations of the 20 up-regulated metabolic network modules in nitrogen limitation identified by AMBIENT. [file 1471-2164-14-436-S3.zip › add3/1851983883936012_add15.pdf]

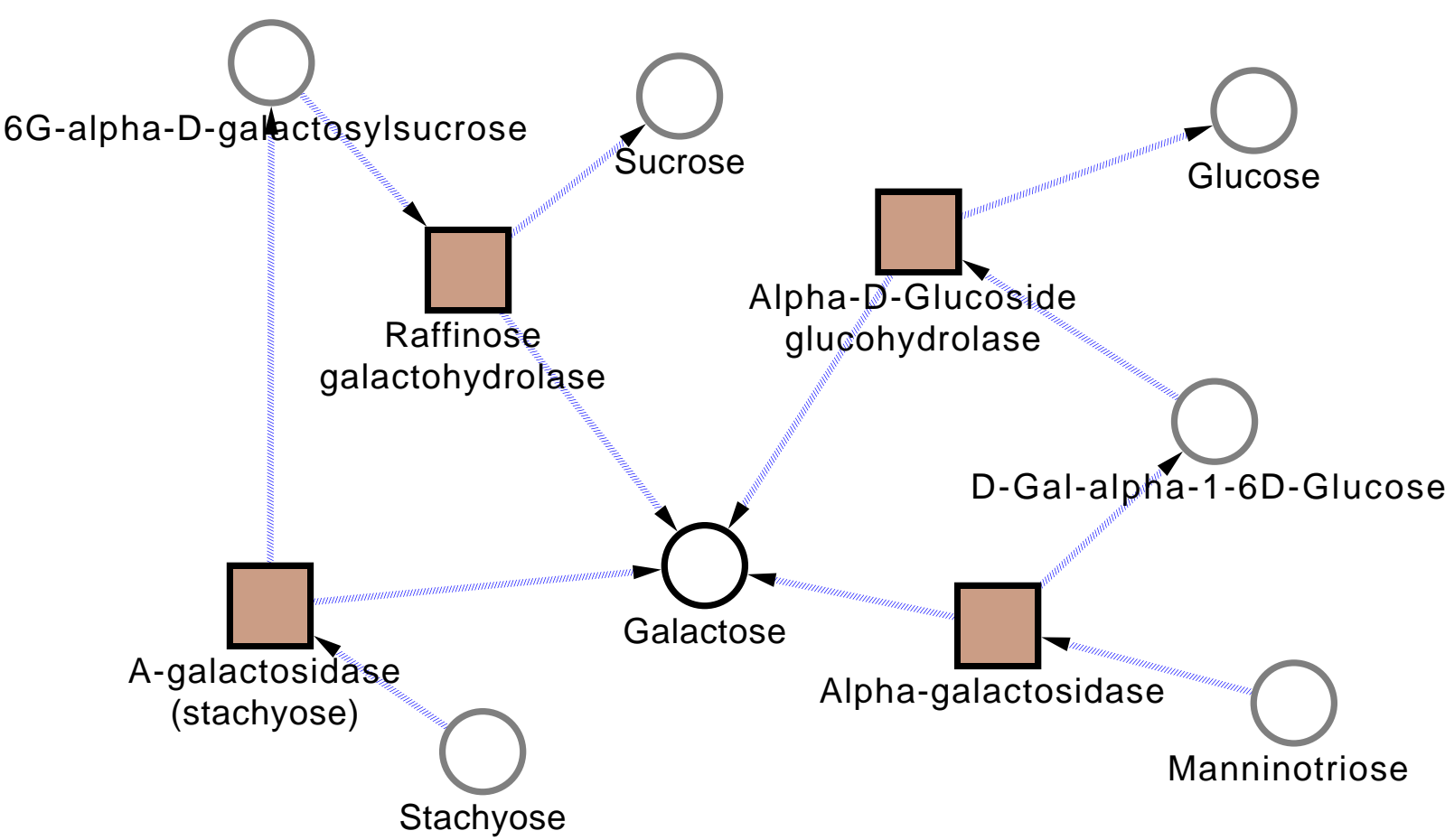

Supplement: Additional file 3 — A zip file containing illustrations of the 20 up-regulated metabolic network modules in nitrogen limitation identified by AMBIENT. [file 1471-2164-14-436-S3.zip › add3/1851983883936012_add16.pdf]

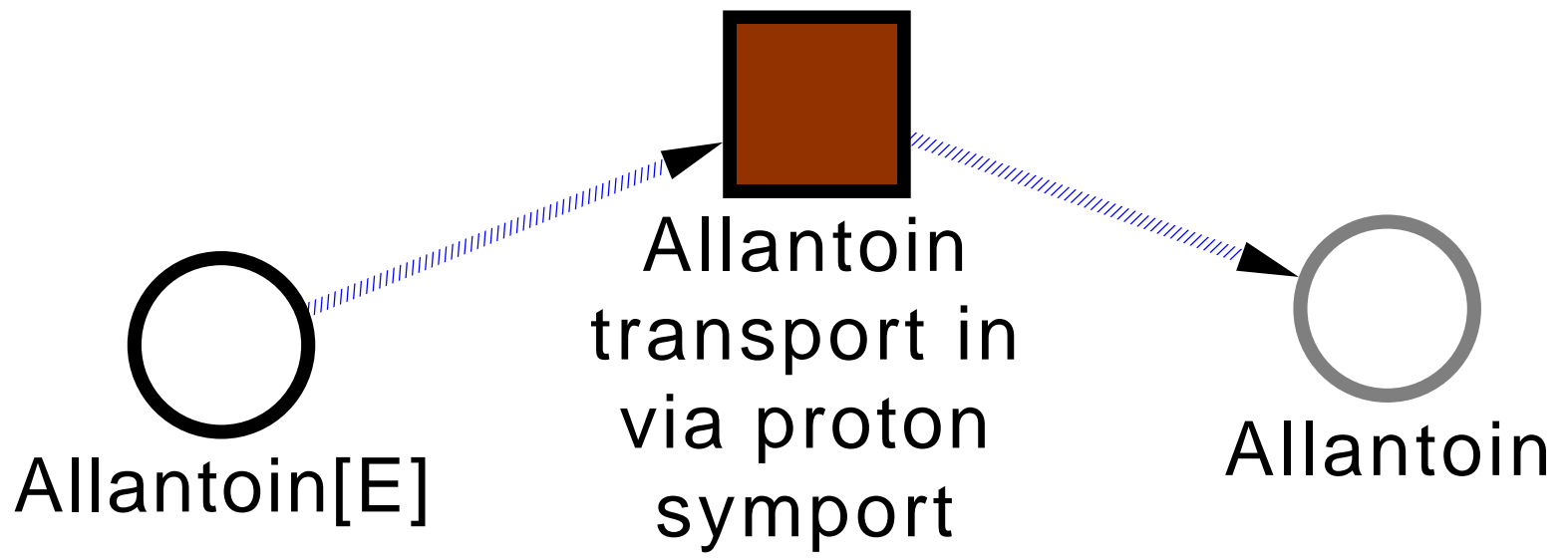

Supplement: Additional file 3 — A zip file containing illustrations of the 20 up-regulated metabolic network modules in nitrogen limitation identified by AMBIENT. [file 1471-2164-14-436-S3.zip › add3/1851983883936012_add17.pdf]

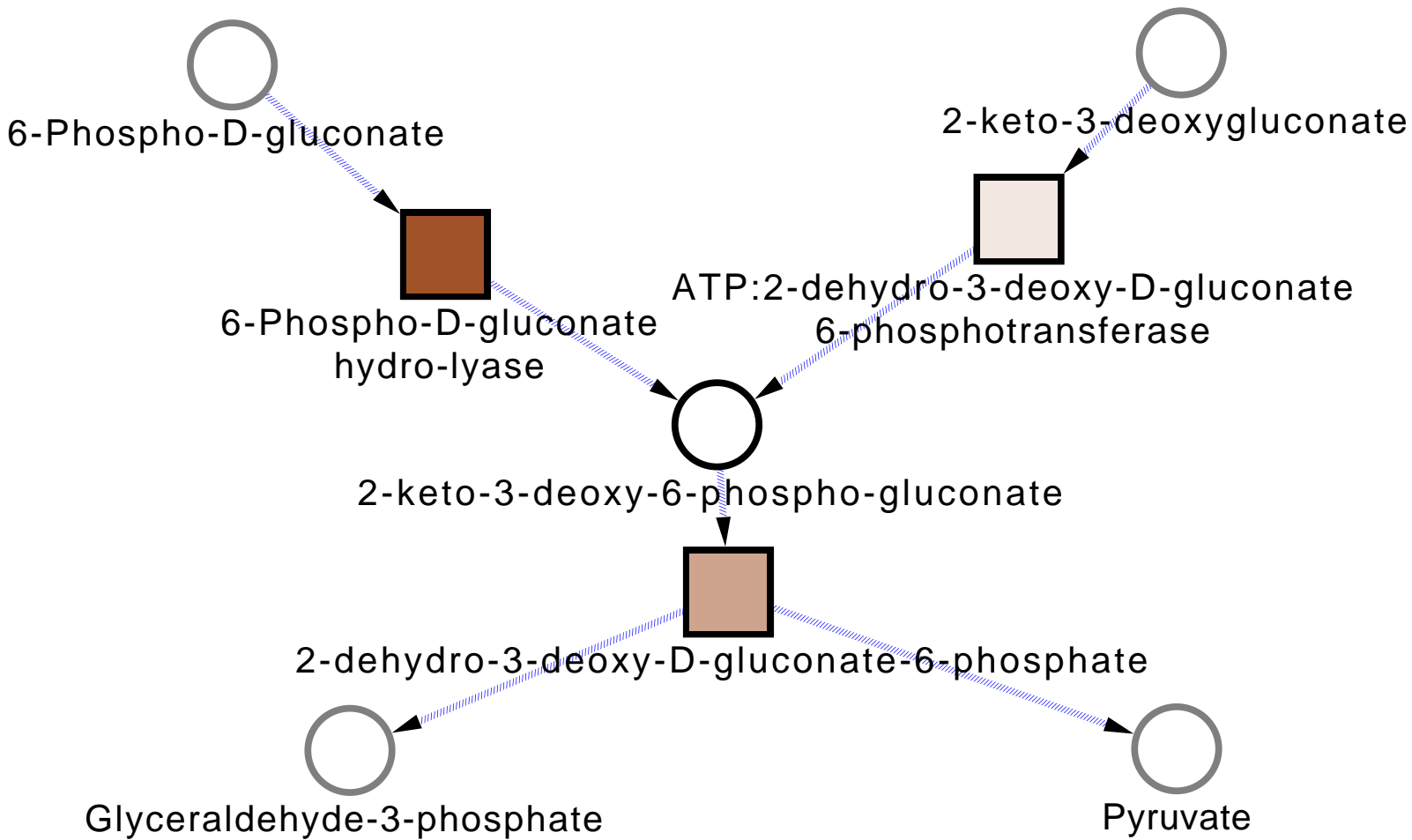

Supplement: Additional file 3 — A zip file containing illustrations of the 20 up-regulated metabolic network modules in nitrogen limitation identified by AMBIENT. [file 1471-2164-14-436-S3.zip › add3/1851983883936012_add18.pdf]

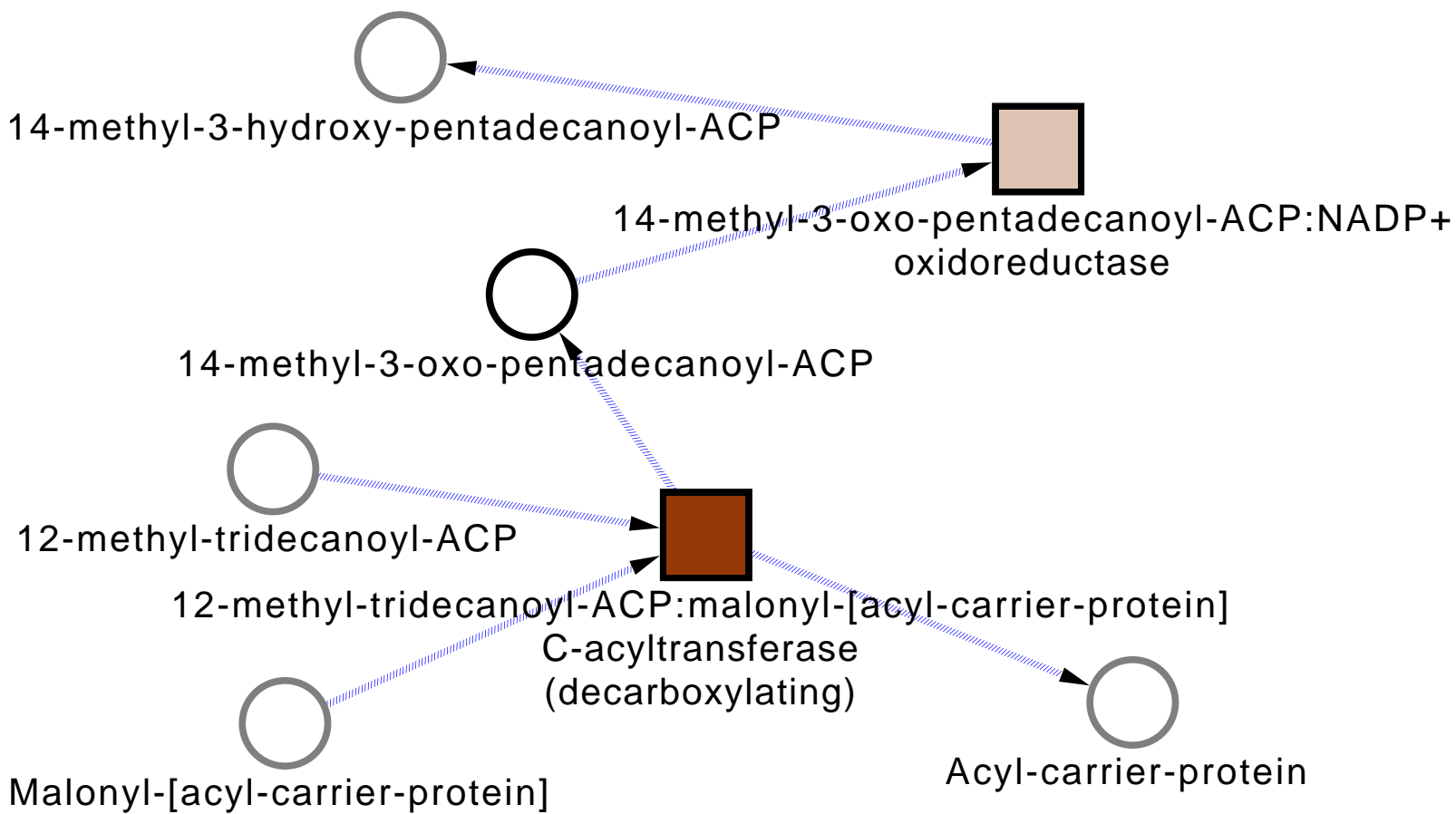

Supplement: Additional file 3 — A zip file containing illustrations of the 20 up-regulated metabolic network modules in nitrogen limitation identified by AMBIENT. [file 1471-2164-14-436-S3.zip › add3/1851983883936012_add19.pdf]

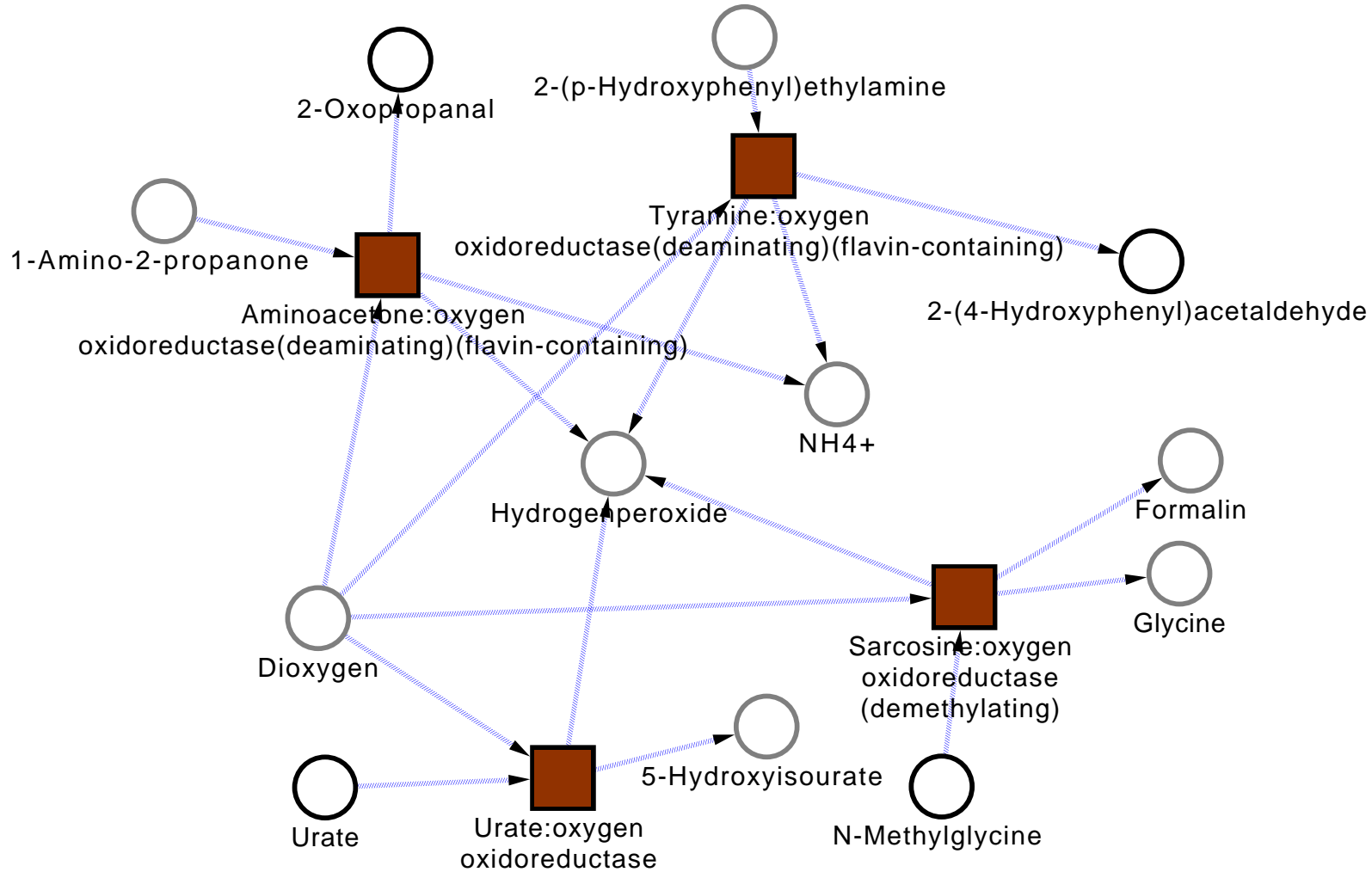

Supplement: Additional file 3 — A zip file containing illustrations of the 20 up-regulated metabolic network modules in nitrogen limitation identified by AMBIENT. [file 1471-2164-14-436-S3.zip › add3/1851983883936012_add20.pdf]

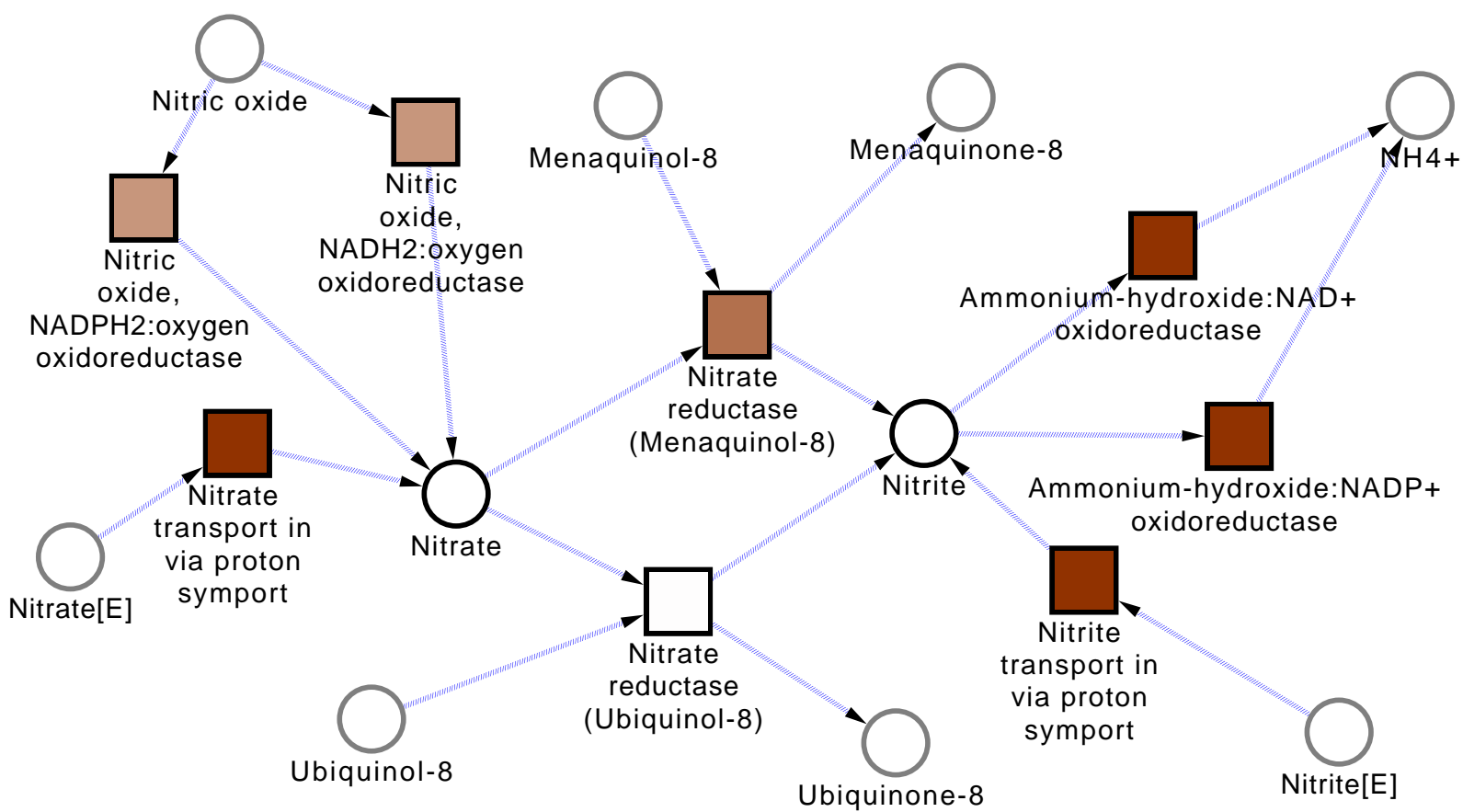

Supplement: Additional file 3 — A zip file containing illustrations of the 20 up-regulated metabolic network modules in nitrogen limitation identified by AMBIENT. [file 1471-2164-14-436-S3.zip › add3/1851983883936012_add4.pdf]

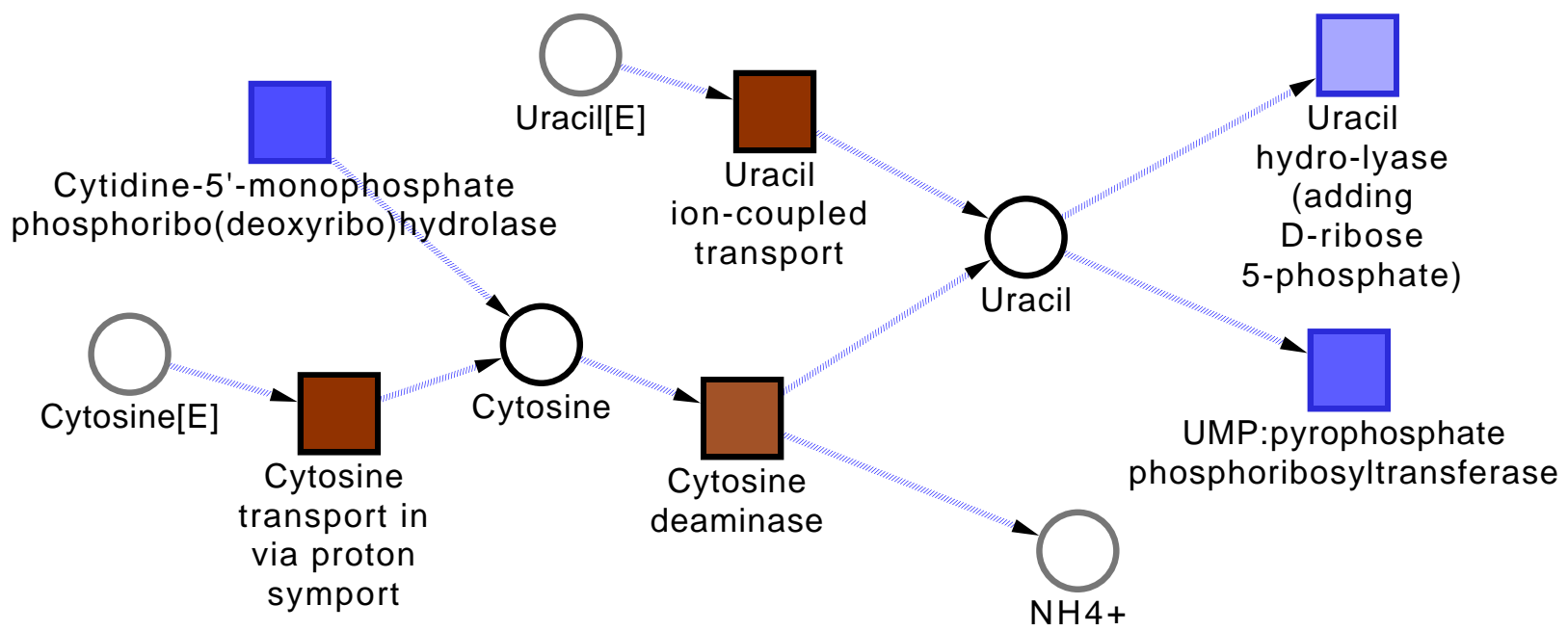

Supplement: Additional file 3 — A zip file containing illustrations of the 20 up-regulated metabolic network modules in nitrogen limitation identified by AMBIENT. [file 1471-2164-14-436-S3.zip › add3/1851983883936012_add5.pdf]

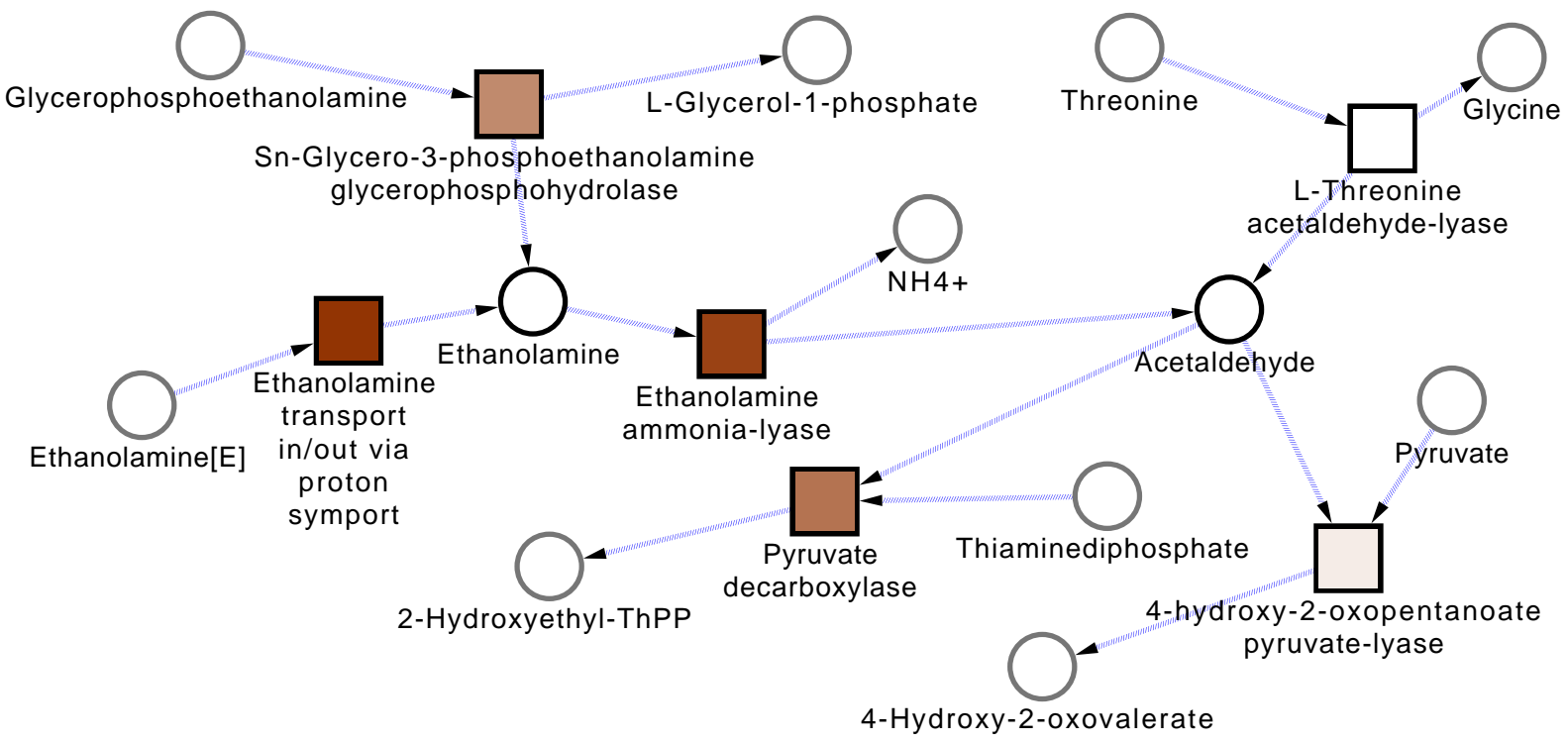

Supplement: Additional file 3 — A zip file containing illustrations of the 20 up-regulated metabolic network modules in nitrogen limitation identified by AMBIENT. [file 1471-2164-14-436-S3.zip › add3/1851983883936012_add6.pdf]

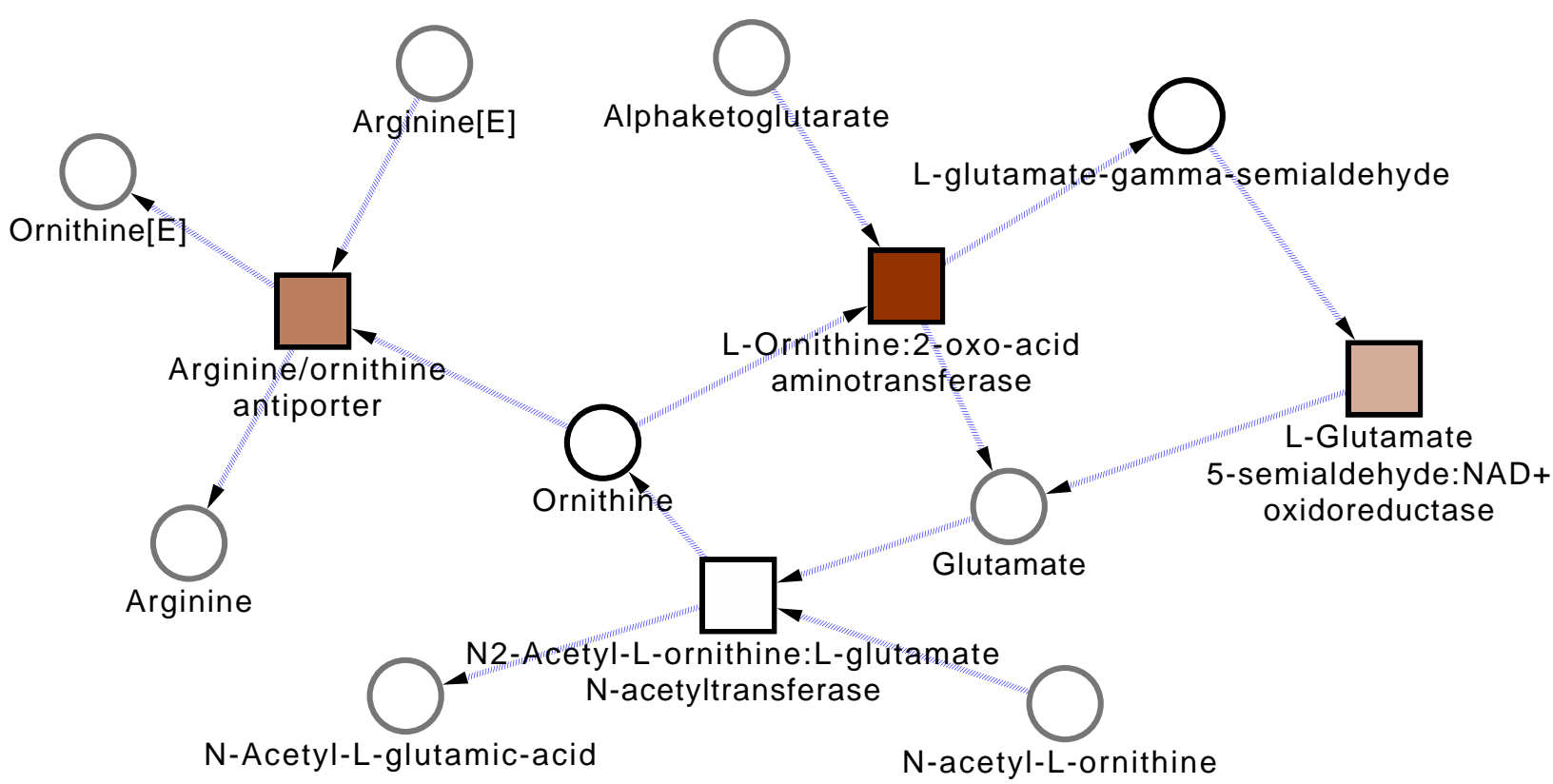

Supplement: Additional file 3 — A zip file containing illustrations of the 20 up-regulated metabolic network modules in nitrogen limitation identified by AMBIENT. [file 1471-2164-14-436-S3.zip › add3/1851983883936012_add7.pdf]

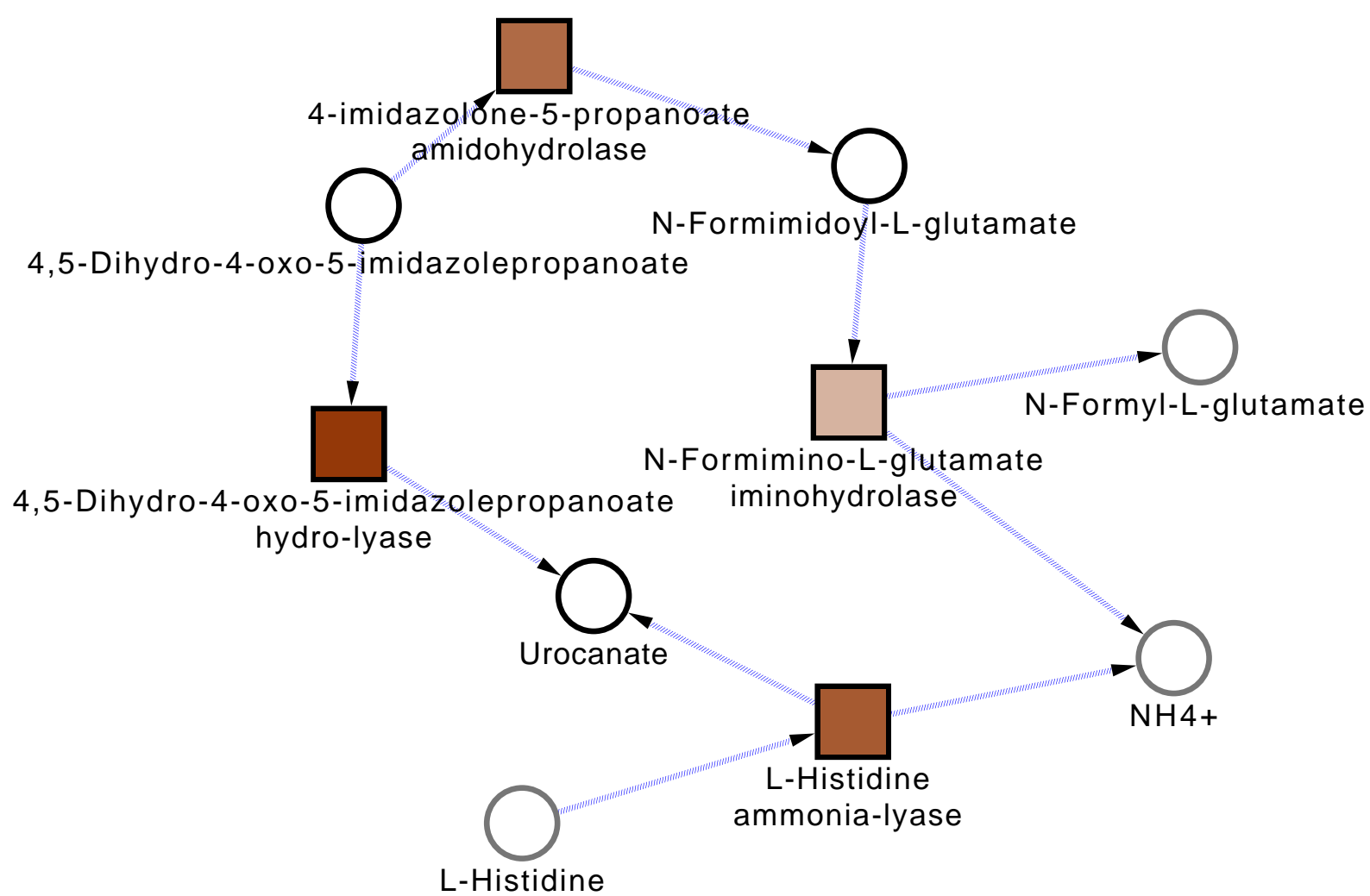

Supplement: Additional file 3 — A zip file containing illustrations of the 20 up-regulated metabolic network modules in nitrogen limitation identified by AMBIENT. [file 1471-2164-14-436-S3.zip › add3/1851983883936012_add8.pdf]

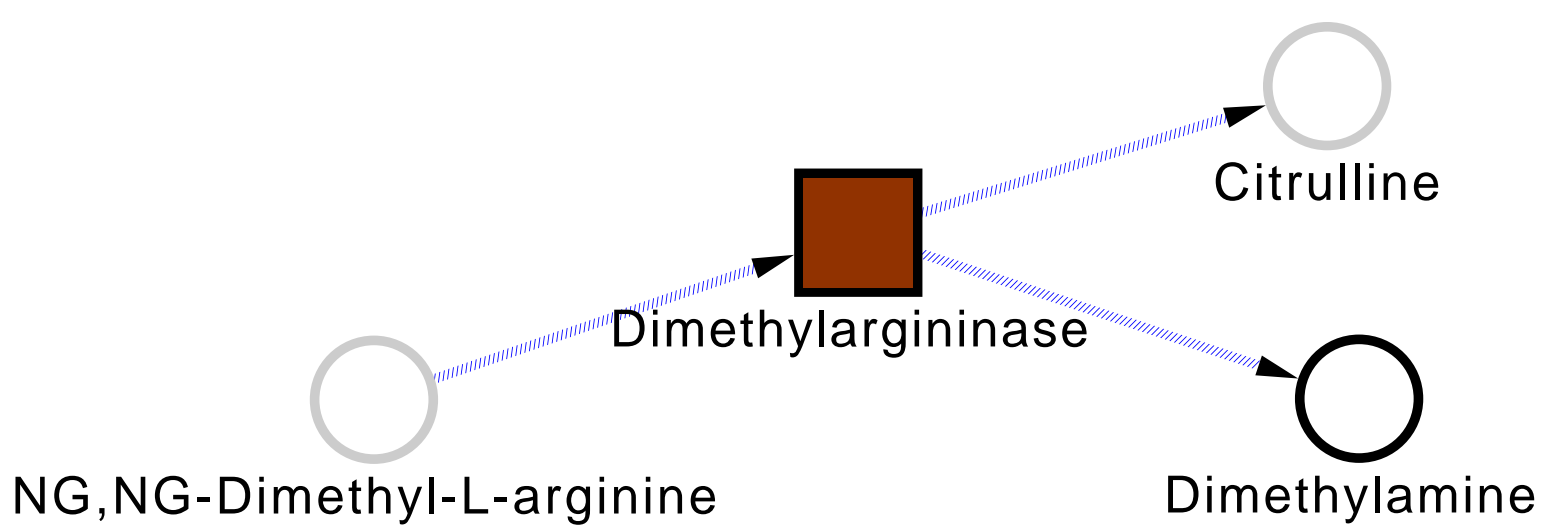

Supplement: Additional file 3 — A zip file containing illustrations of the 20 up-regulated metabolic network modules in nitrogen limitation identified by AMBIENT. [file 1471-2164-14-436-S3.zip › add3/1851983883936012_add9.pdf]

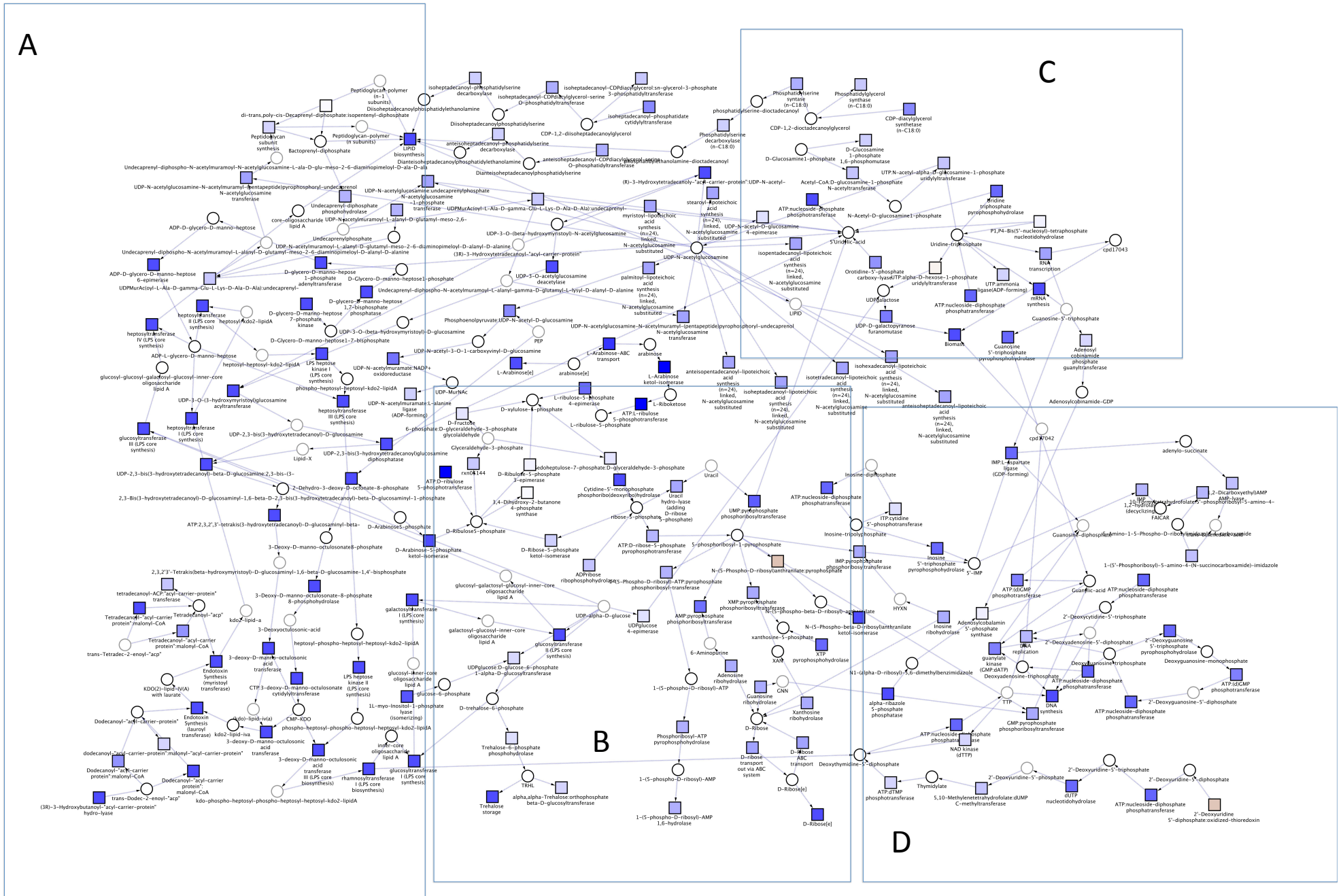

(A) LPS biosynthesis, (B) central carbon metabolism, (C) RNA biosynthesis, and (D) DNA biosynthesis.

Supplement: Additional file 4 — A zip file containing illustrations of the 6 down-regulated metabolic network modules in nitrogen limitation identified by AMBIENT. [file 1471-2164-14-436-S4.zip › add4/1851983883936012_add21.pdf]

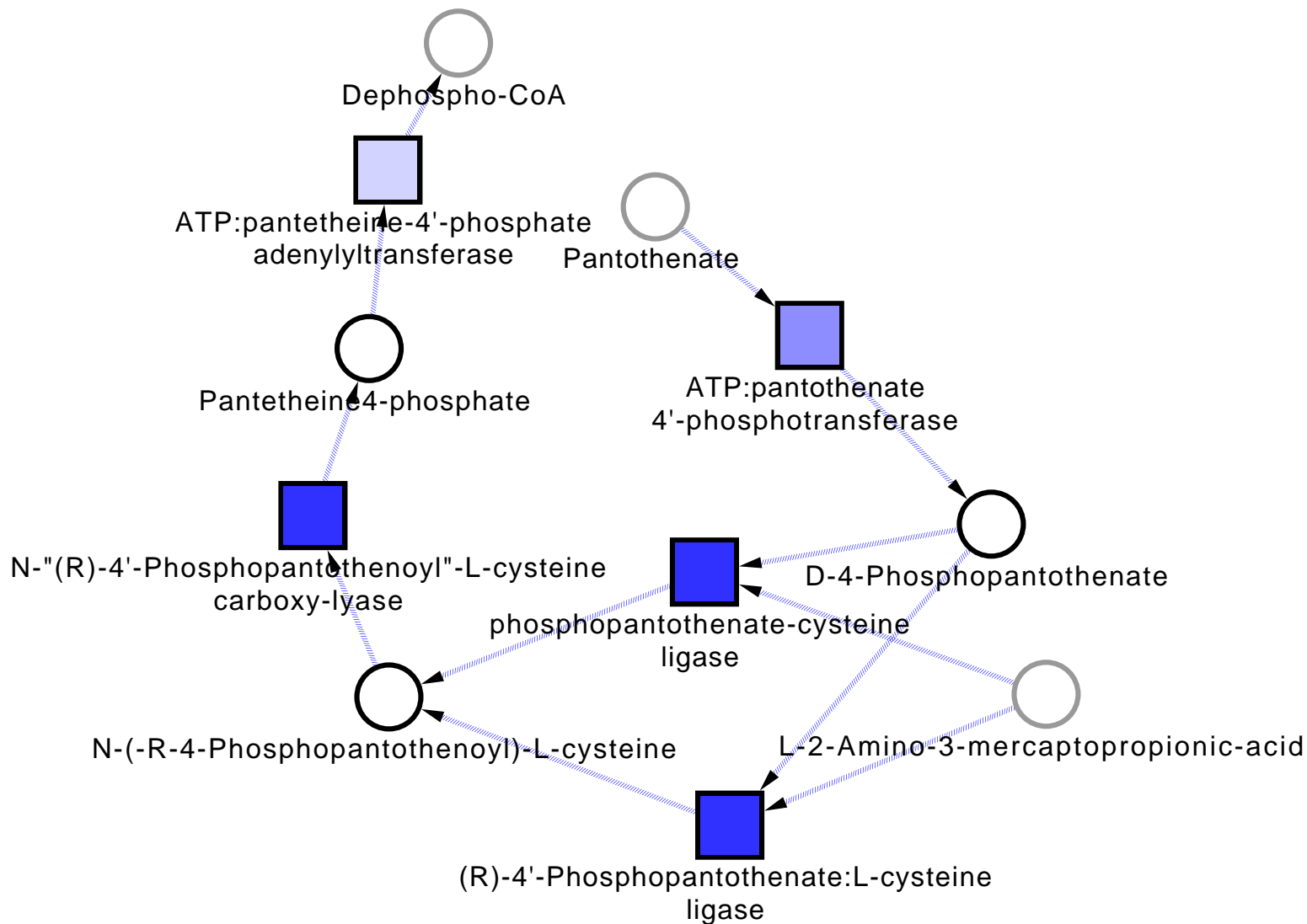

Supplement: Additional file 4 — A zip file containing illustrations of the 6 down-regulated metabolic network modules in nitrogen limitation identified by AMBIENT. [file 1471-2164-14-436-S4.zip › add4/1851983883936012_add22.pdf]

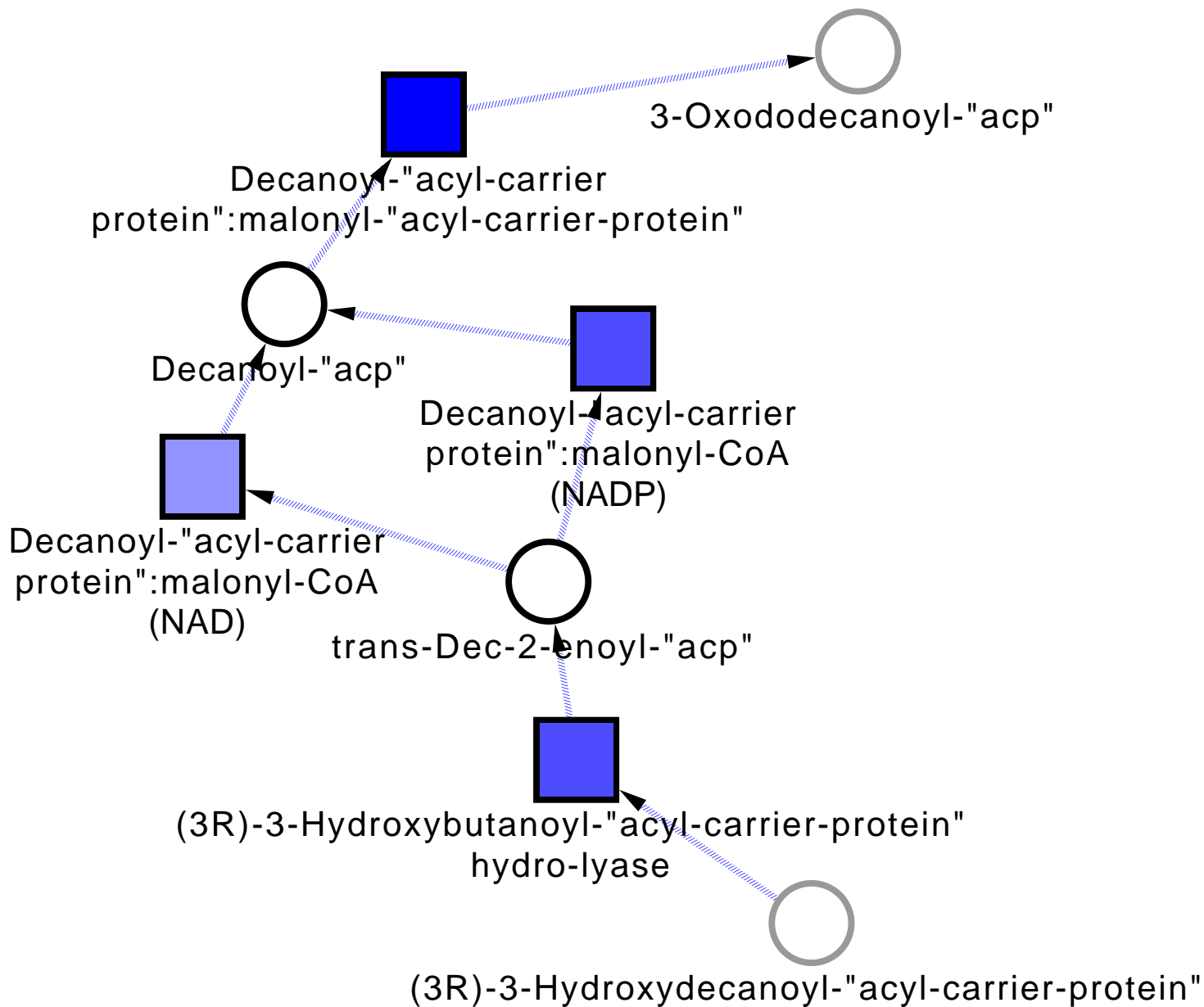

Supplement: Additional file 4 — A zip file containing illustrations of the 6 down-regulated metabolic network modules in nitrogen limitation identified by AMBIENT. [file 1471-2164-14-436-S4.zip › add4/1851983883936012_add23.pdf]

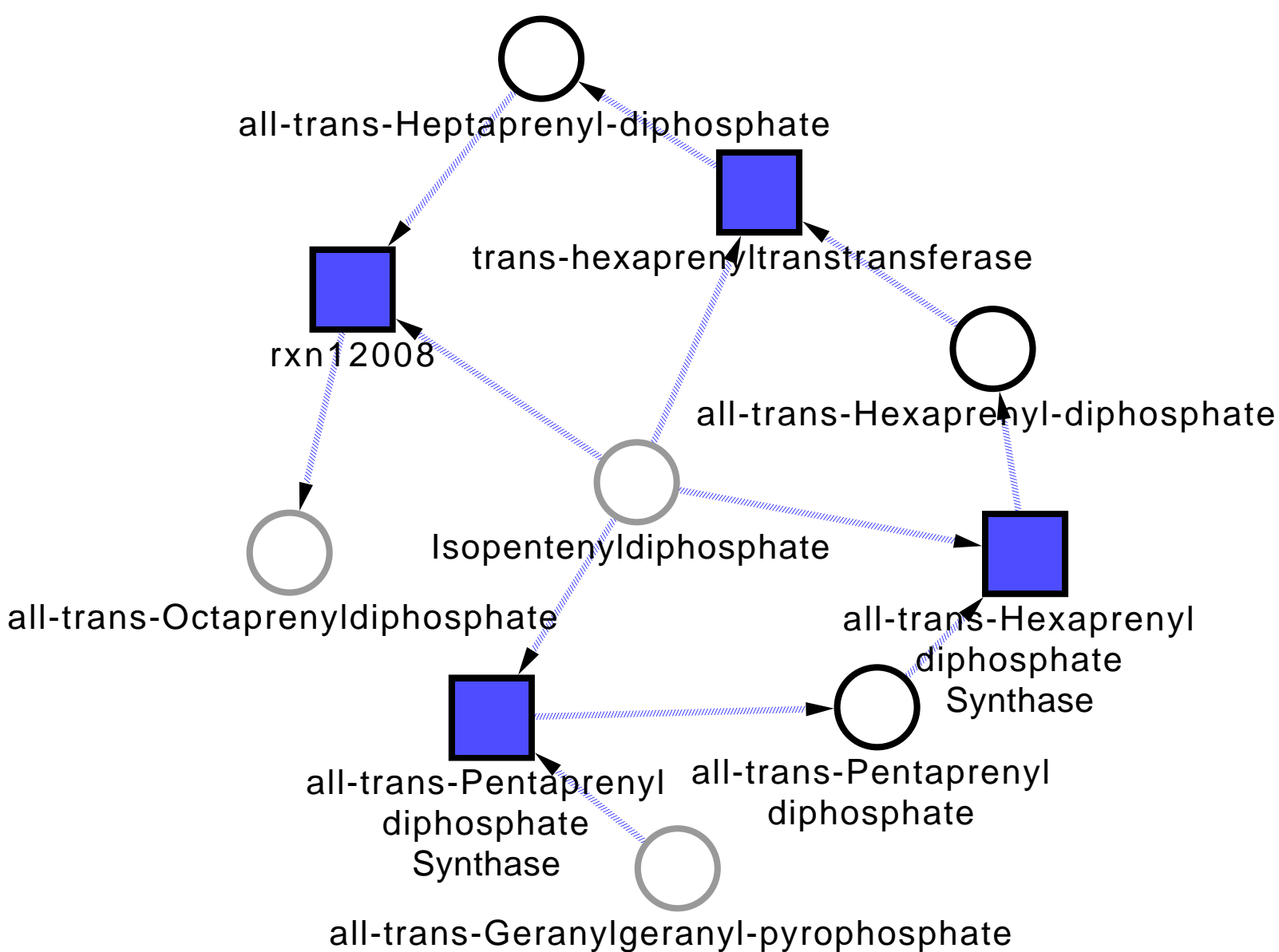

Supplement: Additional file 4 — A zip file containing illustrations of the 6 down-regulated metabolic network modules in nitrogen limitation identified by AMBIENT. [file 1471-2164-14-436-S4.zip › add4/1851983883936012_add24.pdf]

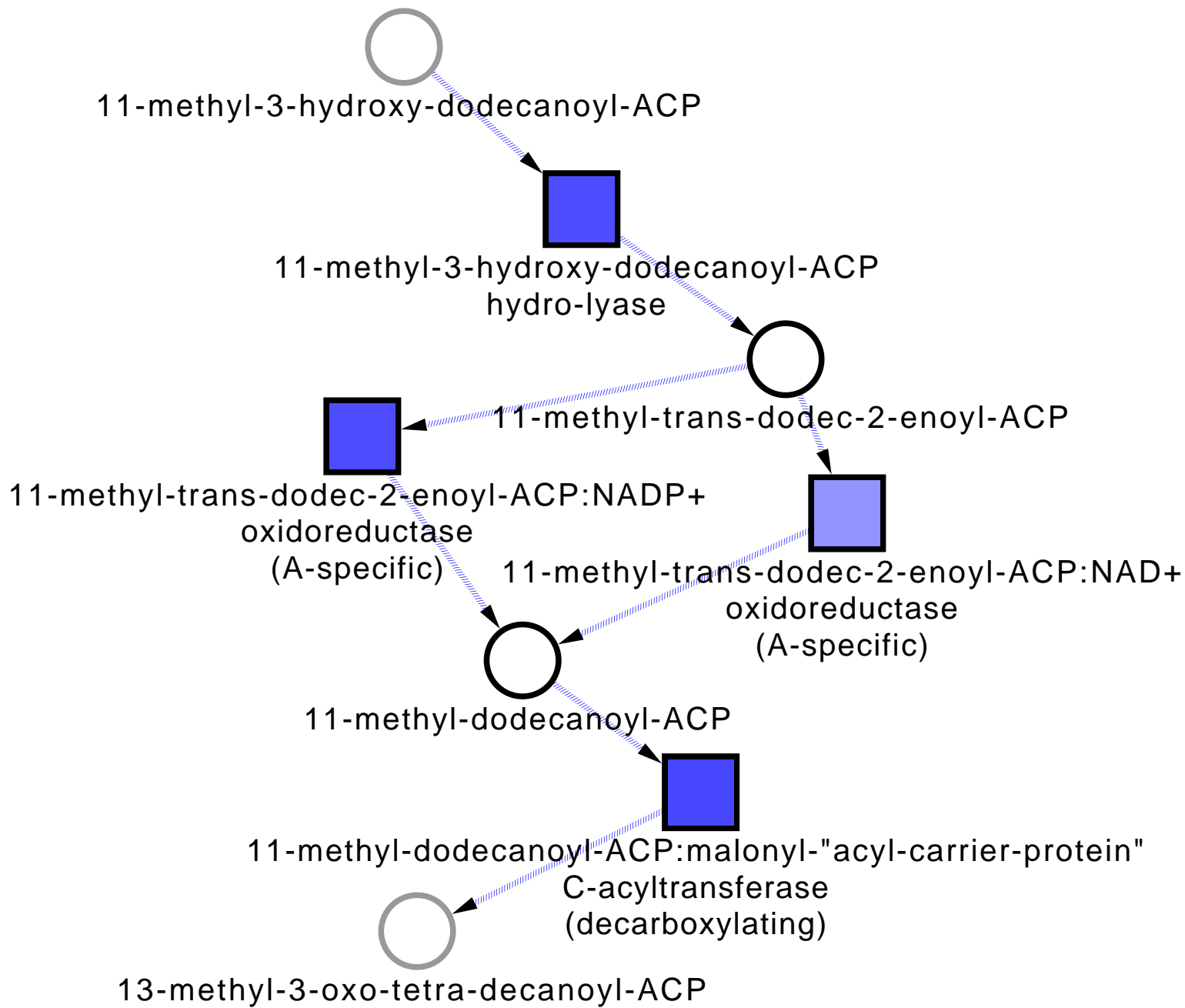

Supplement: Additional file 4 — A zip file containing illustrations of the 6 down-regulated metabolic network modules in nitrogen limitation identified by AMBIENT. [file 1471-2164-14-436-S4.zip › add4/1851983883936012_add25.pdf]

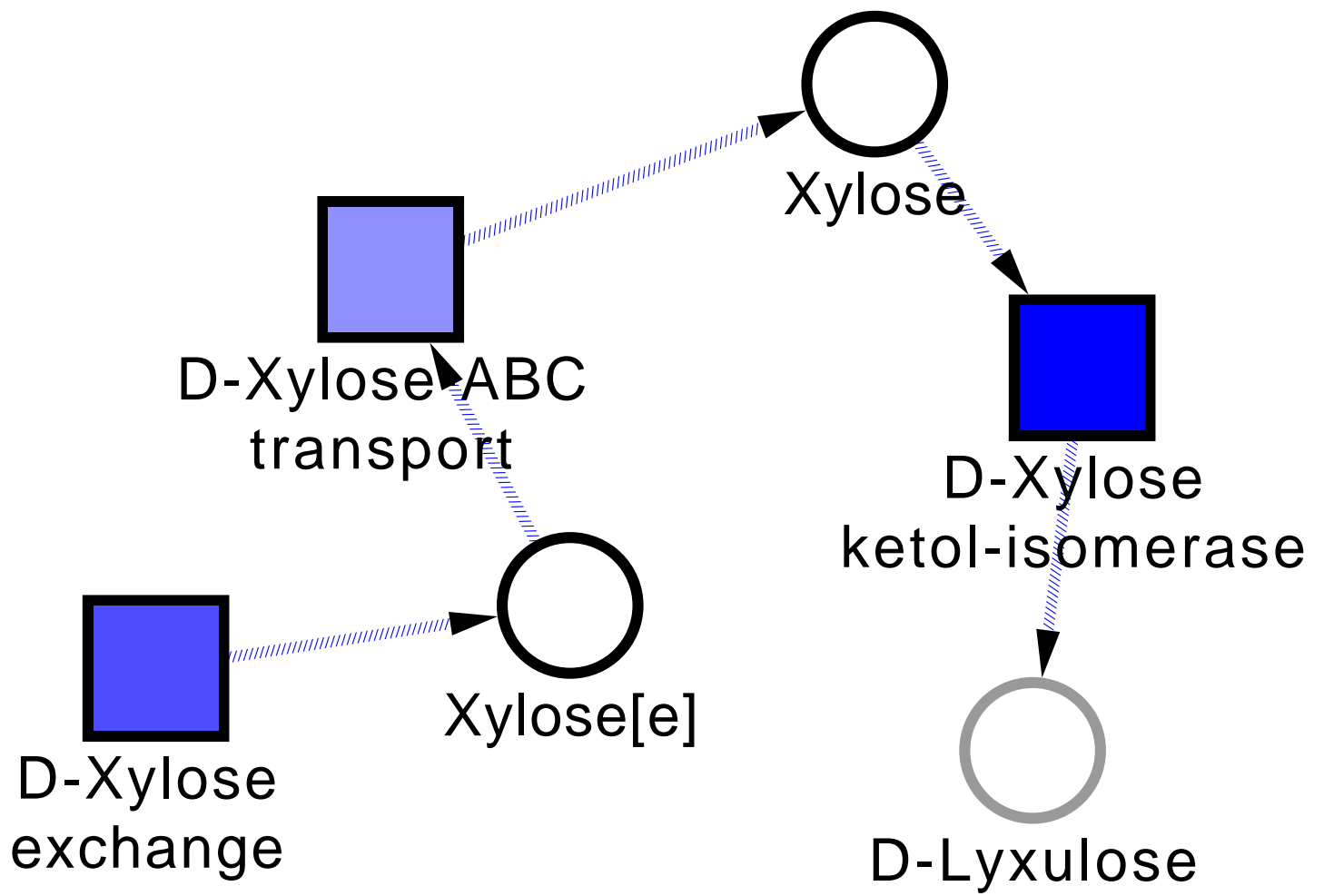

Supplement: Additional file 4 — A zip file containing illustrations of the 6 down-regulated metabolic network modules in nitrogen limitation identified by AMBIENT. [file 1471-2164-14-436-S4.zip › add4/1851983883936012_add26.pdf]

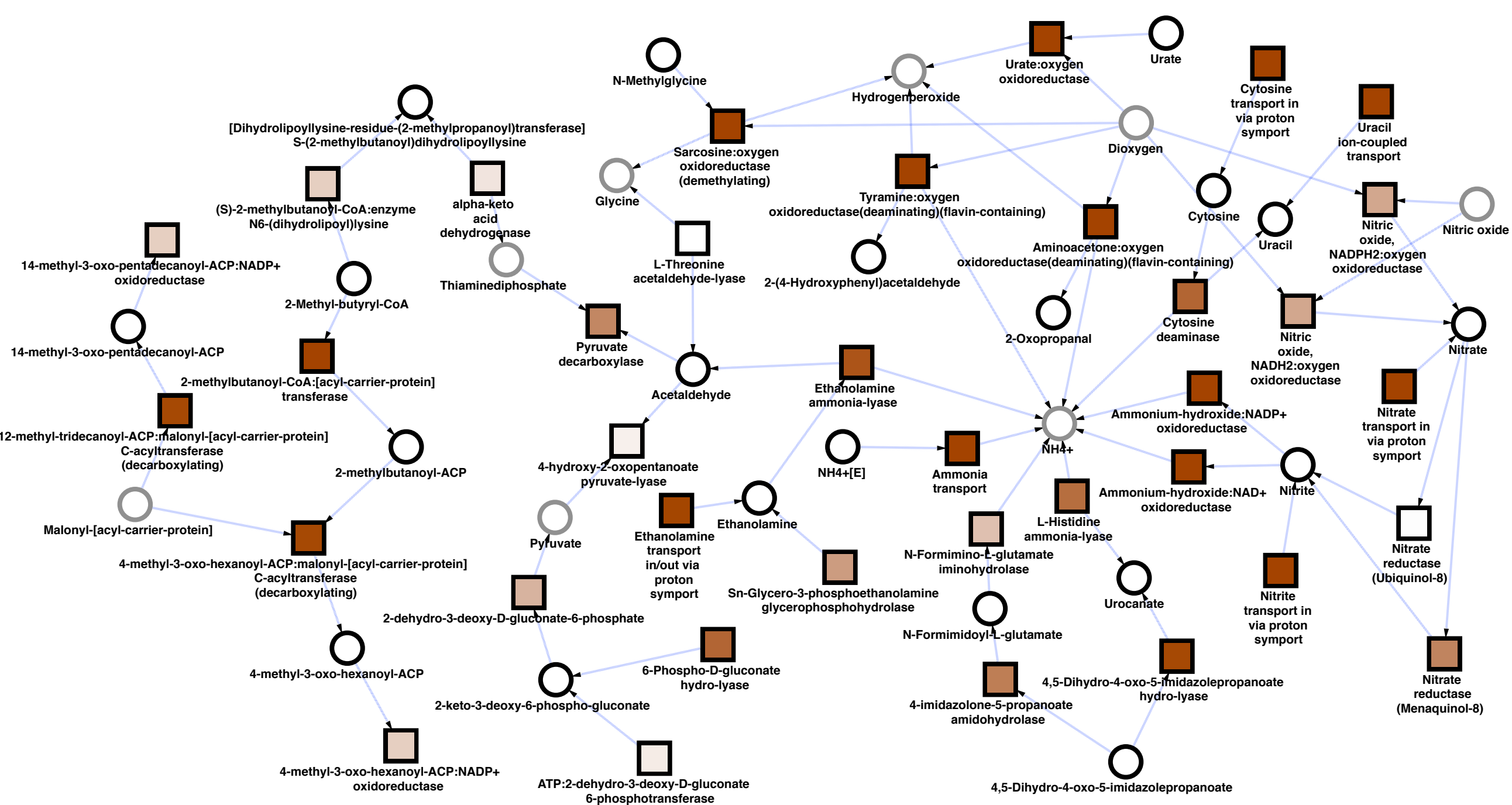

Supplement: Additional file 6 — Up-modules are presented in their metabolic network context, illustrating a cluster of modules around ammonium and around hydrogen peroxide. In S6.1 the nodes in a module are coloured using the original scoring scheme, while in S6.2 the nodes in each module are coloured the same. [file 1471-2164-14-436-S6.zip › add6/1851983883936012_add29.pdf]

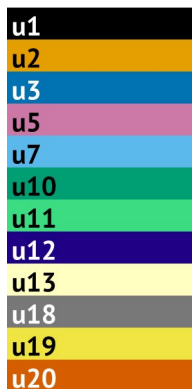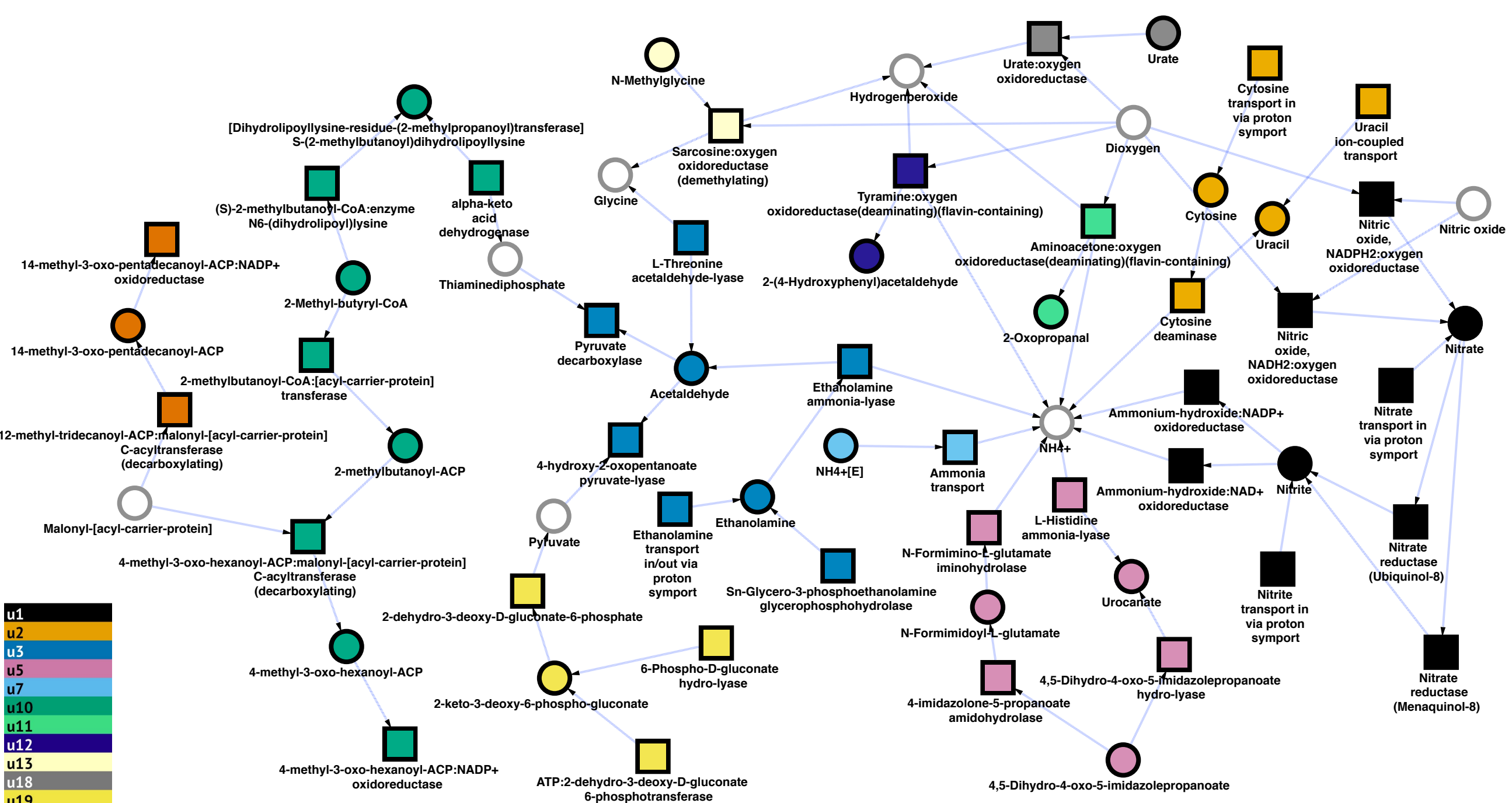

Supplement: Additional file 6 — Up-modules are presented in their metabolic network context, illustrating a cluster of modules around ammonium and around hydrogen peroxide. In S6.1 the nodes in a module are coloured using the original scoring scheme, while in S6.2 the nodes in each module are coloured the same. [file 1471-2164-14-436-S6.zip › add6/1851983883936012_add30.pdf]
