# Supplementary material for: Deciphering the response of Mycobacterium smegmatis to nitrogen stress using bipartite active modules
Source: BMC Genomics. 2013 Jul 2;14:436. doi: 10.1186/1471-2164-14-436 (PMC3706326; doi:10.1186/1471-2164-14-436)
Supplement: Additional file 7 — A table showing the qRT-PCR primers and probe sequences used for the relative quantification of gene expression in M. smegmatis during nitrogen limitation. [file 1471-2164-14-436-S7.docx]

**Table S6.** Custom Taqman *M. smegmatis* gene expression primer and probe sequences used in this study.

| **Gene** | **Forward primer (5’-3’)** | **Reverse primer (5’-3’)** | **FAM Reporter probe (5’-3’)** |
| --- | --- | --- | --- |
| *amt1* (MSMEG6259) | GGCGCAAGCCTCACAAC | AACCGGCCCACAGCAT | CCGCTCACGCTCCTC |
| *amtA* (MSMEG4625) | GCTGGGCCTGATGCTCAT | GTCGCCCGCGTAGATGAT | CTTCACGGGCTTCTTC |
| *amtB* (MSMEG2425) | CACCGTGGTGCACATCAAC | CGGCGTCCGACGATGAT | AAGGGCCGCCATACCG |
| *gltD* (MSMEG3226) | GTCGAGCAGTTGCGTAACC | CCCGGGCACGTCGTT | CCGACGGCGAGCACC |
| *glnD* (MSMEG2427) | CCAGGCGCTCAAGGATCTG | GTCCCGTGCGGTCCA | ATGGTCGCCACTGCCG |
| *glnK* (MSMEG2426) | CCGCGGTGCCGAGTA | CGATGACGTCGACGACCTT | TCGTGCCCAAGGTCC |
| *glnA1* (MSMEG4290) | CAGGCCGAGATCAACTACAAGT | CCATGCGGTGTTCTTGATGATG | ACAGCAGCACATCGTC |
| *sigA* (MSMEG2758) | CGAGAAGGGCGAGAAGCT | CGCCTCCAGCAGATGGTTTT | CAGCGCCGCGACATG |
